# On a Mixed Poisson Liu Regression Estimator for Overdispersed and Multicollinear Count Data

**DOI:** 10.1155/2022/8171461

**Published:** 2022-07-20

**Authors:** Ramajeyam Tharshan, Pushpakanthie Wijekoon

**Affiliations:** ^1^Postgraduate Institute of Science, University of Peradeniya, Peradeniya, Sri Lanka; ^2^Department of Mathematics and Statistics, University of Jaffna, Jaffna, Sri Lanka; ^3^Department of Statistics and Computer Science, University of Peradeniya, Peradeniya, Sri Lanka

## Abstract

The mixed Poisson regression models are commonly employed to analyze the overdispersed count data. However, multicollinearity is a common issue when estimating the regression coefficients by using the maximum likelihood estimator (MLE) in such regression models. To deal with the multicollinearity, a Liu estimator was proposed by Liu (1993). The Poisson-Modification of the Quasi Lindley (PMQL) regression model is a mixed Poisson regression model introduced recently. The primary interest of this paper is to introduce the Liu estimator for the PMQL regression model to mitigate the multicollinearity issue. To estimate the Liu parameter, some exiting methods are used, and the superiority conditions of the new estimator over the MLE and PMQL ridge regression estimator are obtained based on the mean square error (MSE) criterion. A Monte Carlo simulation study and applications are used to assess the performance of the new estimator in the scalar mean square error (SMSE) sense. Based on the simulation study and the results of the applications, it is shown that the PMQL Liu estimator performs better than the MLE and some other existing biased estimators in the presence of multicollinearity.

## 1. Introduction

The Poisson regression model is a commonly used statistical method for analyzing the count response variable [1]. One disadvantage of this model is that it is an overdispersion issue that is common in the real-world applications of actuarial, engineering, biomedical, and economic sciences. The overdispersion occurs when the conditional variance of the count response variable exceeds the conditional mean of the count response variable. In this context, the index of dispersion (variance-to-mean ratio) is greater than one. To tackle this issue in the Poisson regression model, researchers have proposed several mixed Poisson regression models. The standard mixed Poisson distribution is obviously the negative binomial (NB)/Poisson-gamma regression model introduced by Greenwood and Yule [2]. However, the NB distribution fails to fit well for a count data with a higher value of the index of dispersion and long right-tail behavior. Then, the regression model based on NB is not a good choice for such a count response variable. As an alternative to the NB regression model, several mixed Poisson regression models are in the literature. However, most of the probability mass functions (pmfs) of these mixed Poisson distributions are not in an explicit form. Some notable examples of such regression models are the Poisson-Inverse Gaussian regression model [3] and the Poisson-Inverse gamma regression model [4]. This algebraic intractability in such distributions leads to computational complexity, and their regression models are limited in practice.

In the last decade, several researchers have highlighted mixed Poisson distributions obtained by mixing the Poisson and Lindley family of distributions due to their explicit form of the pmf and work efficiency. The Lindley family of distributions are two-component mixtures. Some notable such mixed Poisson distributions are Poisson–Lindley distribution, Generalized Poisson–Lindley distribution, Poisson-generalized Lindley distribution, Poisson-Quasi Lindley distribution, and Poisson-weighted Lindley distribution, proposed by Sankaran [5], Mahmoudi and Zakerzadeh [6], Wongrin and Bodhisuwan [7], Grine and Zeghdoudi [8], and Atikankul et al. [9], respectively. They may have the flexibility to capture various ranges of horizontal symmetries, right-tail behaviors, and variance-to-mean ratios based on their mixing distributions [10–12]. However, the literature on their regression models is rather limited. Some of the most relevant works cited are the Generalized Poisson–Lindley (GPL) regression model derived by Wongrin and Bodhisuwan [13] and the Poisson-Quasi Lindley (PQL) regression model obtained by Altun [14].

Tharshan and Wijekoon [15] obtained a new Lindley family of distributions named the Modification of Quasi Lindley (MQL) distribution. Its probability density function (pdf) is given as

(1) $$\begin{array}{c} f_{Y}\left(y;\theta ,\alpha ,\delta \right)=\frac{\theta e^{-\theta y}}{\left(\alpha^{3}+1\right)\Gamma \left(\delta \right)}\left(\Gamma \left(\delta \right)\alpha^{3}+{\left(\theta y\right)}^{\delta -1}\right);\;y>0;\;\theta >0,\;\alpha^{3}>-1,\;\delta >0, \end{array}$$

where *α* and *δ* are shape parameters, *θ* is a scale parameter, and *y* is the respective random variable bounded to (0, *∞*). Equation (1) presents the mixture of exponential (*θ*) and gamma (*δ*, *θ*) distributions with the mixing proportion, *p* = (*α*^3^/*α*^3^ + 1). By mixing the Poisson and the MQL, Tharshan and Wijekoon [16] derived the Poisson-Modification of the Quasi Lindley (PMQL) distribution. Its explicit form of the pmf, and some other important statistics are given in Section 2. Authors have shown that the PMQL distribution is an overdispersed distribution, and it has the flexibility to capture the various ranges of horizontal symmetry, right-tail heaviness, and variance-to-mean ratio. Then, by using a reparameterization technique, the same authors [17] derived its regression model to predict the overdispersed count responses with a set of linear independent covariates based on the generalized linear model (GLM) approach. Further, in their paper, it is shown that the PMQL regression model performs better than the NB, GPL, and PQL regression models. More details of this regression model are given in Section 2.The traditional estimator to estimate the unknown regression coefficients of the PMQL regression model is the maximum likelihood estimator (MLE_(PMQL)_), where the solutions of the nonlinear equations with respect to the regression coefficients are found by applying an iterative weighted least square (IWLS) algorithm. However, the MLE_(PMQL)_ is unstable, and its variance is inflated when the covariates are linearly correlated since it is a GLM. It leads to difficulty in having a valid statistical inference. This problem is commonly known as multicollinearity by Frisch [18]. To overcome the multicollinearity problem in the PMQL regression model, Tharshan and Wijekoon [19] adopted the ridge regression estimator in the PMQL regression model (RE_(PMQL)_). The authors have shown that the RE_(PMQL)_ performs better than the MLE_(PMQL)_ when multicollinearity exists. Further, they recommended some ridge parameter estimation methods for the RE_(PMQL)_. The ridge regression estimator was suggested by Hoerl and Kennard [20] for the ordinary linear regression model, and it was extended to GLM by Segerstedf [21]. The ordinary linear regression model is defined as

(2) $$\begin{array}{c} y=X\beta +\varepsilon , \end{array}$$

where *y* is an *n* × 1 vector of observations on a response variable *Y*, *β* is a (*p* + 1) × 1 vector of unknown regression coefficients, *X* is the known design matrix of order *n* × (*p* + 1) with *p*-dimensional covariates, and *ε* is an *n* × 1 vector of errors with *E*(*ε*) = 0 and Var(*ε*) = *σ*^2^*I*_*n*_. Further, its unknown regression coefficients are estimated by the ordinary least square estimator, which is defined as

(3) $$\begin{array}{c} {\hat{\beta }}_{\text{OLSE}}={\left(X^{\prime }X\right)}^{-1}X^{\prime }y. \end{array}$$

Even though the ridge estimator is an efficacious one, its drawback is that it includes a complicated nonlinear function of the ridge parameter *k*, which is bounded to (0, *∞*). Therefore, Kejian [22] proposed a biased estimator named the Liu estimator for the ordinary linear regression model (LE_(OR)_), which is a linear function of the Liu parameter *d* bounded to (0,1) by modifying the ordinary least square estimator $\left({\hat{\beta }}_{\text{OLSE}}\right)$. The Liu estimator is defined in the ordinary linear regression as

(4) $$\begin{array}{c} {\hat{\beta }}_{{\text{LE}}_{\left(\text{OR}\right)}}={\left(X^{\prime }X+I\right)}^{-1}\left(X^{\prime }X+dI\right){\hat{\beta }}_{\text{OLSE}}, \end{array}$$

where *I* is the identity matrix of order (*p*+1) × (*p*+1) and *d* ∈ (0,1) is the Liu parameter.

Due to the advantageous property of the Liu estimator (linear function with respect to the *d*) over the ridge estimator, the Liu estimator has been considered by several researchers for different GLMs. Mansson et al. [23] discussed some improved Liu estimators for the Poisson regression model; Mansson et al. [24] adopted the Liu estimator in the logit regression model; Mansson [25] developed a Liu estimator for the negative binomial regression model; Siray et al. [26] introduced a restricted Liu estimator for the logistic regression model; Wu [27] derived a modified restricted Liu estimator in logistic regression model; Kurtoğlu and Özkale [28] proposed the Liu estimator for the generalized linear regression models and discussed an application on gamma distributed response variable; Türkan and Özel [29] proposed the Jackknifed estimators for the negative binomial regression model; Wu et al. [30] introduced a restricted almost unbiased Liu estimator for the logistic regression model; Varathan and Wijekoon [31] obtained a logistic Liu estimator under stochastic linear restrictions; Qasim et al. [32] proposed some new Liu parameter estimators for Poisson regression model; Li et al. [33] obtained stochastic restricted Liu estimator in logistic regression model; and Omer et al. [34] developed Liu estimators for the zero-inflated Poisson regression model. We may note that the Liu estimator for the regression model of a mixed Poisson distribution is rather limited in the literature.

This paper adopts the Liu estimator in the PMQL regression model to combat the multicollinearity. Further, we adhere to some possible estimation methods to estimate the Liu parameter *d* for the PMQL Liu regression estimator (LE_(*PMQL*)_) based on the works carried out by Hoerl and Kennard [20], Kibria [35], and Khalaf and Shukur [36]. Then, the performance of the MLE_(*PMQL*)_, RE_(*PMQL*)_, and LE_(*PMQL*)_ will be compared in terms of the scalar mean square error (SMSE) criterion by using an extensive Monte Carlo simulation study. Finally, a simulated data set and a real-world example will be considered to illustrate the benefits of the Liu estimator for the PMQL regression model in handling the overdispersion and multicollinearity issues.

The rest of the paper is organized as follows: Section 2 discusses the PMQL regression model and its regression coefficients estimator. We present the LE_(PMQL)_, mean square error (MSE) properties of the LE_(PMQL)_, conditions that the LE_(PMQL)_ is superior to the MLE_(PMQL)_ and the RE_(PMQL)_, and possible Liu parameter estimators for the LE_(PMQL)_ in Section 3.  Section 4 designs a Monte Carlo simulation study and discusses the results of the simulation study.  Section 5 gives a simulated data set and real data applications in order to illustrate the applicability of the PMQL Liu regression model. Finally, the conclusion of the paper is given in Section 6.

## 2. PMQL Regression Model

In this section, we present the PMQL regression model and its regression coefficients estimation.

The PMQL distribution [16] is a resultant distribution or unconditional distribution by assuming that the Poisson parameter follows the MQL distribution. The pdf of the MQL distribution is given in equation (1). The probability mass function of the PMQL distribution is given as

(5) $$\begin{array}{c} f_{Y}\left(y\right)=\frac{\theta }{y!\left(\alpha^{3}+1\right){\left(1+\theta \right)}^{y+\delta }\Gamma \left(\delta \right)}\left(\Gamma \left(\delta \right)\Gamma \left(y+1\right)\alpha^{3}{\left(1+\theta \right)}^{\delta -1}+\theta^{\delta -1}\Gamma \left(y+\delta \right)\right);\;y=0,1,2,\ldots ,\;\theta >0,\;\delta >0,\;\alpha^{3}>-1, \end{array}$$

where *y* is the respective random variable and represents the total counts of an experiment. Its mean and variance are given

(6) $$\begin{array}{c} E\left(Y\right)=\frac{\alpha^{3}+\delta }{\left(\alpha^{3}+1\right)\theta }=\mu , \end{array}$$

(7) $$\begin{array}{c} \text{Var}\left(Y\right)=\mu +\mu^{2}\left(\frac{\alpha^{3}\left(\alpha^{3}+2+\delta \left(\delta -1\right)\right)+\delta }{{\left(\alpha^{3}+\delta \right)}^{2}}\right), \end{array}$$

respectively. Equation (5) represents a two-component mixture of geometric (*θ*/1+*θ*) and negative binomial (*δ*, 1/1+*θ*) with the mixing proportion, *p*=(*α*^3^/*α*^3^+1). Further, it possesses to be unimodal and bimodal distributions and overdispersed. The authors have shown that it has the potentiality to accommodate various horizontal symmetry, right-tail behaviors, and index of dispersion for overdispersed count data.

Let *y*_1_, *y*_2_,…, *y*_*n*_ be the random sample of *n* observations from the PMQL distribution. The link between *p*-dimensional covariates and the mean responses *y* was taken as

(8) $$\begin{array}{c} \eta_{i}=g\left(\mu_{i}\right)=\log\left(\mu_{i}\right)=\sum_{j=0}^{p}\beta_{j}x_{ij}=x_{i}^{\prime }\beta ,\;i=1,2,\ldots ,n, \end{array}$$

where *x*_*i*_′ = (1, *x*_*i*1_, *x*_*i*2_,…, *x*_*ip*_) is the vector of *i*^*th*^ row of the known design matrix *X* supplemented with a 1 in front for the intercept, *β*′ = (*β*_0_, *β*_1_,…, *β*_*p*_) is a vector of unknown regression coefficients of order (*p* + 1) × 1 with intercept, and *α* and *δ* are overdispersion parameters. To approach the GLM, the PMQL distribution was reparametrized based on the relationship between *μ* and *θ* given in equation (6) for a given set of *α* and *δ* values and the link between *μ* and *p*-dimensional covariates given in equation (8).

**Figure 1:**
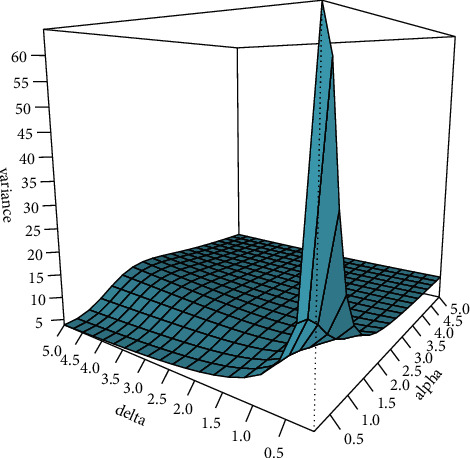
The variance function of the PMQL regression model at *μ*=2.50 for different values of *α* and *δ*.

That is, by substituting *θ*_*i*_=(*α*^3^+*δ*)/(*α*^3^+1)exp(*x*_*i*_′*β*),  *i*=1,2,…, *n* in equation (5), the pmf of the *y*_*i*_ for a given set of covariates *x*_*i*_′ was obtained as

(9) $$\begin{array}{c} f\left(y_{i}|x_{i}^{\prime }\right)=\frac{{\left(\left(\alpha^{3}+1\right)\exp\left(x_{i}^{\prime }\beta \right)\right)}^{y_{i}}\left(\alpha^{3}+\delta \right)\left.\left(\Gamma \left(\delta \right)\right.\Gamma \left(y_{i}+1\right)\alpha^{3}A_{i}^{\delta -1}+{\left(\alpha^{3}+\delta \right)}^{\delta -1}\Gamma \left(y_{i}+\delta \right)\right)}{y_{i}!\left(\alpha^{3}+1\right)A_{i}^{y_{i}+\delta }\Gamma \left(\delta \right)}, \end{array}$$

where *A*_*i*_=((*α*^3^+1)exp(*x*_*i*_′*β*)+(*α*^3^+*δ*)),  *i*=1,2,…, *n*. The conditional mean and variance of the regression model are given:

(10) $$\begin{array}{c} E\left(Y_{i}|x_{i}^{\prime }\right)=\exp\left(x_{i}^{\prime }\beta \right),\\ \text{Var}\left(Y_{i}|x_{i}^{\prime }\right)=\exp\left(x_{i}^{\prime }\beta \right)+{\left(\exp\left(x_{i}^{\prime }\beta \right)\right)}^{2}\left(\frac{\alpha^{3}\left(\alpha^{3}+2+\delta \left(\delta -1\right)\right)+\delta }{{\left(\alpha^{3}+\delta \right)}^{2}}\right), \end{array}$$

respectively.  Figure 1 depicts the surface plots of the variance function of the PMQL regression model at *μ*=2.5 for different values of *α* and *δ*. From Figure 1, it can be observed that the variance as a function of *α* or *δ* is not a monotonic function, and it is high for small values of *α* and *δ*.

The estimation of the unknown regression coefficients is commonly estimated by maximizing the following log-likelihood function of its pmf given in equation (9):

(11) $$\begin{array}{c} \ell \left(\beta ,\alpha ,\delta |y,x\right)=\sum_{i=1}^{n}y_{i}\log\left(\left(\alpha^{3}+1\right)\exp\left(x_{i}^{\prime }\beta \right)\right)+n\;\log\left(\alpha^{3}+\delta \right)-\sum_{i=1}^{n}\log\left(y_{i}!\right)-n\;\log\left(\alpha^{3}+1\right)-n\;\log\left(\Gamma \left(\delta \right)\right)\\ +\sum_{i=1}^{n}\left(\Gamma \left(\delta \right)\Gamma \left(y_{i}+1\right)\alpha^{3}A_{i}^{\delta -1}+{\left(\alpha^{3}+\delta \right)}^{\delta -1}\Gamma \left(y_{i}+\delta \right)\right)-\sum_{i=1}^{n}\left(y_{i}+\delta \right)\log\left(A_{i}\right). \end{array}$$

The score function of the vector of regression coefficients *β* is given as

(12) $$\begin{array}{c} S\left(\beta \right)=\frac{\partial l\left(\beta ,\alpha ,\delta |y,x\right)}{\partial \beta }=\sum_{i=1}^{n}y_{i}x_{i}-\sum_{i=1}^{n}\frac{\left(y_{i}+\delta \right)\left(\alpha^{3}+1\right)\exp\left(x_{i}^{\prime }\beta \right)x_{i}}{A_{i}}\\ +\sum_{i=1}^{n}\frac{\Gamma \left(\delta \right)\Gamma \left(y_{i}+1\right)\alpha^{3}\left(\delta -1\right)A_{i}^{\delta -2}\left(\alpha^{3}+1\right)\exp\left(x_{i}^{\prime }\beta \right)x_{i}}{\Gamma \left(\delta \right)\Gamma \left(y_{i}+1\right)\alpha^{3}A_{i}^{\delta -1}+{\left(\alpha^{3}+\delta \right)}^{\delta -1}\Gamma \left(y_{i}+\delta \right)}. \end{array}$$

Since equation (12) is nonlinear in *β*, one can use the iteratively weighted least square (IWLS) algorithm (Fisher scoring method) [16] to obtain the maximum likelihood (ML) estimates. Let *β*^(*s* − 1)^ be the estimated value of *β* by the ML method with (*s* − 1) iterations. Then, the Fisher scoring method can be written as

(13) $$\begin{array}{c} \beta^{\left(s\right)}=\beta^{\left(s-1\right)}+I^{-1}\left(\beta^{\left(s-1\right)}\right)S\left(\beta^{\left(s-1\right)}\right), \end{array}$$

where *I*(*β*^(*s* − 1)^) is a (*p* + 1) × (*p* + 1) Fisher information matrix and the *S*(*β*^(*s* − 1)^) is the score function of the regression coefficients calculated at *β*^(*s* − 1)^. In the final step of the IWLS algorithm, ${\hat{\beta }}_{{\text{MLE}}_{\left(\text{PMQL}\right)}}$ is obtained as

(14) $$\begin{array}{c} {\hat{\beta }}_{{\text{MLE}}_{\left(\text{PMQL}\right)}}={\left(X^{\prime }\hat{W}X\right)}^{-1}X^{\prime }\hat{W}\hat{z}, \end{array}$$

where

(15) $$\begin{array}{c} \hat{W}=\text{diag}\left(\frac{1}{{g^{\prime }\left(\left(\hat{\mu_{i}}\right)\right)}^{2}\text{Var}\left(\hat{\mu_{i}}\right)}\right)=\text{diag}\left(\frac{\hat{\mu_{i}{\left(\alpha^{3}+\delta \right)}^{2}}}{{\left(\alpha^{3}+\delta \right)}^{2}+\hat{\mu_{i}\left(\alpha^{3}\left(\alpha^{3}+2+\delta \left(\delta -1\right)\right)+\delta \right)}}\right), \end{array}$$

$\hat{z}$ is a vector, and its *i*^*th*^ element is given as $g\hat{\left(\mu_{i}\right)}+\hat{\left(y_{i}-\hat{\mu_{i}}\right)g^{\prime }\left(\mu_{i}\right)}=\log\left(\hat{\mu_{i}}\right)+\left(y_{i}-\hat{\mu_{i}}\right)/\hat{\mu_{i}}$.

The asymptotic covariance matrix of this estimator is given as

(16) $$\begin{array}{c} \text{Cov}\left({\hat{\beta }}_{{\text{MLE}}_{\left(\text{PMQL}\right)}}\right)={\left(X^{\prime }\hat{W}X\right)}^{-1}, \end{array}$$

and the asymptotic MSE and SMSE of this estimator are given as [16]

(17) $$\begin{array}{c} \text{MSE}\left({\hat{\beta }}_{{\text{MLE}}_{\left(\text{PMQL}\right)}}\right)={\left(X^{\prime }\hat{W}X\right)}^{-1} \end{array}$$

(18) $$\begin{array}{c} \text{SMSE}\left({\hat{\beta }}_{{\text{MLE}}_{\left(\text{PMQL}\right)}}\right)=\sum_{j=1}^{p+1}\frac{1}{\lambda_{j}}, \end{array}$$

respectively, where *λ*_*j*_ is the *j*^*th*^ eigenvalue of the matrix $X^{\prime }\hat{W}X$.

When the covariates are highly correlated, the weighted matrix of cross-product $X^{\prime }\hat{W}X$ is ill-conditioned, and this matrix will have some smaller eigenvalues. We can observe that the SMSE $\left({\hat{\beta }}_{{\text{MLE}}_{\left(\text{PMQL}\right)}}\right)$ given in equation (18) can easily be inflated for smaller eigenvalues. In this situation, it is very hard to have a valid inference of whether the estimated regression coefficients are significant or not.

## 3. The PMQL Liu Regression Estimator

Note that the PMQL regression model is a GLM. Then, following the Liu estimator for the GLMs, which was proposed by Kurtoğlu and Özkale [28] for the GLMs based on the IWLS algorithm, we define the Liu estimator for the PMQL regression model to mitigate the multicollinearity issue as

(19) $$\begin{array}{c} {\hat{\beta }}_{{\text{LE}}_{\left(\text{PMQL}\right)}}^{\left(s\right)}={\left(X^{\prime }\hat{W}\left(\beta^{\left(s-1\right)}\right)X+I\right)}^{-1}\left(X^{\prime }\hat{W}\left(\beta^{\left(s-1\right)}\right)X+dI\right){\hat{\beta }}_{{\text{MLE}}_{\left(PMQL\right)}}^{\left(s\right)}, \end{array}$$

where ${\hat{\beta }}_{{\text{LE}}_{\left(\text{PMQL}\right)}}^{\left(s\right)}$ is the estimated value of the *β* by the Liu estimator with the *s* iterations, $\hat{W}\left(\beta^{\left(s-1\right)}\right)$ is the weighted matrix evaluated at *β*^(*s* − 1)^, and ${\hat{\beta }}_{{\text{MLE}}_{\left(\text{PMQL}\right)}}^{\left(s\right)}$ is the estimated value of the *β* by the maximum likelihood method with the *s* iterations. In the final step of the IWLS algorithm, ${\hat{\beta }}_{{\text{LE}}_{\left(\text{PMQL}\right)}}$ can be obtained as

(20) $$\begin{array}{c} {\hat{\beta }}_{{\text{LE}}_{\left(\text{PMQL}\right)}}={\left(X^{\prime }\hat{W}X+I\right)}^{-1}\left(X^{\prime }\hat{W}X+dI\right){\hat{\beta }}_{{\text{MLE}}_{\left(\text{PMQL}\right)}}=L_{d}{\hat{\beta }}_{{\text{MLE}}_{\left(PMQL\right)}}, \end{array}$$

where *d*(0 < *d* < 1) is the Liu parameter, *I* is a (*p* + 1) × (*p* + 1) identity matrix, and $L_{d}={\left(X^{\prime }\hat{W}X+I\right)}^{-1}\left(X^{\prime }\hat{W}X+dI\right)$. Note that, if *d* = 1, then ${\hat{\beta }}_{{\text{MLE}}_{\left(\text{PMQL}\right)}}={\hat{\beta }}_{{\text{LE}}_{\left(\text{PMQL}\right)}}$.

Asymptotic properties of the PMQL Liu regression estimator are as follows:

(21) $$\begin{array}{c} E\left({\hat{\beta }}_{{\text{LE}}_{\left(\text{PMQL}\right)}}\right)=L_{d}\beta ,\\ \text{Cov}\left({\hat{\beta }}_{{\text{LE}}_{\left(\text{PMQL}\right)}}\right)=L_{d}\text{Cov}\left({\hat{\beta }}_{{\text{MLE}}_{\left(\text{PMQL}\right)}}\right)L_{d}^{\prime }\\ ={\left(X^{\prime }\hat{W}X+I\right)}^{-1}\left(X^{\prime }\hat{W}X+dI\right){\left(X^{\prime }\hat{W}X\right)}^{-1}\left(X^{\prime }\hat{W}X+dI\right)\prime {\left(X^{\prime }\hat{W}X+I\right)}^{-1}\\ ={\left(X^{\prime }\hat{W}X+I\right)}^{-1}\left(X^{\prime }\hat{W}X+dI\right)\left(I+d{\left(X^{\prime }\hat{W}X\right)}^{-1}\right){\left(X^{\prime }\hat{W}X+I\right)}^{-1}\\ =L_{d}\left(I+d{\left(X^{\prime }\hat{W}X\right)}^{-1}\right){\left(X^{\prime }\hat{W}X+I\right)}^{-1}, \end{array}$$

and then the asymptotic bias and MSE are given as

(22) $$\begin{array}{c} \text{Bias}\left({\hat{\beta }}_{{\text{LE}}_{\left(\text{PMQL}\right)}}\right)=E\left({\hat{\beta }}_{{\text{LE}}_{\left(\text{PMQL}\right)}}\right)-\beta \\ =\left(L_{d}-I\right)\beta \\ =\left({\left(X^{\prime }\hat{W}X+I\right)}^{-1}\left(X^{\prime }\hat{W}X+dI\right)-I\right)\beta \\ =\left(d-1\right){\left(X^{\prime }\hat{W}X+I\right)}^{-1}\beta ,\\ \text{MSE}\left({\hat{\beta }}_{{\text{LE}}_{\left(\text{PMQL}\right)}}\right)=E\left(\left({\hat{\beta }}_{{\text{LE}}_{\left(\text{PMQL}\right)}}-\beta \right){\left({\hat{\beta }}_{{\text{LE}}_{\left(\text{PMQL}\right)}}-\beta \right)}^{\prime }\right)\\ =\text{Cov}\left({\hat{\beta }}_{{\text{LE}}_{\left(\text{PMQL}\right)}}\right)+\text{Bias}\left({\hat{\beta }}_{{\text{LE}}_{\left(\text{PMQL}\right)}}\right){\text{Bias}}^{\prime }\left({\hat{\beta }}_{{\text{LE}}_{\left(\text{PMQL}\right)}}\right)\\ =L_{d}\left(I+d{\left(X^{\prime }\hat{W}X\right)}^{-1}\right){\left(X^{\prime }\hat{W}X+I\right)}^{-1}+{\left(d-1\right)}^{2}{\left(X^{\prime }\hat{W}X+I\right)}^{-1}\beta \beta^{\prime }{\left(X^{\prime }\hat{W}X+I\right)}^{-1}, \end{array}$$

respectively. Now, we derive the asymptotic SMSE of the estimator as

(23) $$\begin{array}{c} \text{SMSE}\left({\hat{\beta }}_{{\text{LE}}_{\left(\text{PMQL}\right)}}\right)=\text{trace}\left(\text{MSE}\left({\hat{\beta }}_{{\text{LE}}_{\left(\text{PMQL}\right)}}\right)\right)\\ =\text{trace}\left(\text{Cov}\left({\hat{\beta }}_{{\text{LE}}_{\left(\text{PMQL}\right)}}\right)\right)+{\text{Bias}}^{\prime }\left({\hat{\beta }}_{{\text{LE}}_{\left(\text{PMQL}\right)}}\right)\text{Bias}\left({\hat{\beta }}_{{\text{LE}}_{\left(\text{PMQL}\right)}}\right)\\ =\text{trace}\left({\left(X^{\prime }\hat{W}X\right)}^{-1}L_{d}^{\prime }L_{d}\right)+{\left(d-1\right)}^{2}\beta^{\prime }{\left(X^{\prime }\hat{W}X+I\right)}^{-2}\beta \\ =\text{trace}\left({\left(X^{\prime }\hat{W}X\right)}^{-1}{\left(X^{\prime }\hat{W}X+dI\right)}^{\prime }{\left(X^{\prime }\hat{W}X+I\right)}^{-2}\left(X^{\prime }\hat{W}X+dI\right)\right)+{\left(d-1\right)}^{2}\beta^{\prime }{\left(X^{\prime }\hat{W}X+I\right)}^{-2}\beta . \end{array}$$

Let us define an orthogonal matrix Γ whose columns are the normalized eigenvectors of the matrix $X^{\prime }\hat{W}X$, a vector *α* = Γ′*β*, and a diagonal matrix $\Lambda =\text{diag}\left(\lambda_{1},\lambda_{2},\ldots ,\lambda_{p+1}\right)=\Gamma^{\prime }X^{\prime }\hat{W}X\Gamma$. Then, the asymptotic SMSE can be written by using the spectral decomposition as

(24) $$\begin{array}{c} \text{SMSE}\left({\hat{\beta }}_{{\text{LE}}_{\left(PMQL\right)}}\right)=\text{trace}\left(\Gamma \Gamma^{\prime }{\left(X^{\prime }\hat{W}X\right)}^{-1}\Gamma \Gamma^{\prime }{\left(X^{\prime }\hat{W}X+dI\right)}^{\prime }\Gamma \Gamma^{\prime }{\left(X^{\prime }\hat{W}X+I\right)}^{-2}\Gamma \Gamma^{\prime }\left(X^{\prime }\hat{W}X+dI\right)\right)\\ +{\left(d-1\right)}^{2}\beta^{\prime }\Gamma \Gamma^{\prime }{\left(X^{\prime }\hat{W}X+I\right)}^{-2}\Gamma \Gamma^{\prime }\beta \\ =\text{trace}\left(\Lambda^{-1}{\left(\Lambda +dI\right)}^{\prime }{\left(\Lambda +I\right)}^{-2}\left(\Lambda +dI\right)\right)+{\left(d-1\right)}^{2}\beta^{\prime }\Gamma {\left(\Lambda +I\right)}^{-2}\Gamma^{\prime }\beta \\ =\sum_{j=1}^{p+1}\frac{{\left(\lambda_{j}+d\right)}^{2}}{\lambda_{j}{\left(\lambda_{j}+1\right)}^{2}}+{\left(d-1\right)}^{2}\sum_{j=1}^{p+1}\frac{\alpha_{j}^{2}}{{\left(\lambda_{j}+1\right)}^{2}}\\ =\text{term I}+\text{ term II},\text{say,} \end{array}$$

respectively, where *α*_*j*_(*j* = 1,2,…, *p* + 1) is the *j*^*th*^ element of Γ′*β* and term I and term II are the total variance of regression coefficient estimates and squared bias, respectively.

### 3.1. MSE Properties of the PMQL Liu Regression Estimator

In this subsection, we discuss the MSE properties of the Liu estimator for the PMQL regression model. Further, we make a comparison of ${\hat{\beta }}_{{\text{LE}}_{\left(\text{PMQL}\right)}}$ with the existing estimators such as ${\hat{\beta }}_{{\text{MLE}}_{\left(\text{PMQL}\right)}}$ and ${\hat{\beta }}_{{\text{RE}}_{\left(\text{PMQL}\right)}}$ to show the superiority of ${\hat{\beta }}_{{\text{LE}}_{\left(\text{PMQL}\right)}}$ under different conditions in the MSE sense.

Let us define Δ which is the SMSE differences of ${\hat{\beta }}_{{\text{MLE}}_{\left(\text{PMQL}\right)}}$ and ${\hat{\beta }}_{{\text{LE}}_{\left(\text{PMQL}\right)}}$. By using equations (18) and (24), we get

(25) $$\begin{array}{c} \Delta =\text{SMSE}\left({\hat{\beta }}_{{\text{MLE}}_{\left(\text{PMQL}\right)}}\right)-\text{SMSE}\left({\hat{\beta }}_{{\text{LE}}_{\left(\text{PMQL}\right)}}\right)=\sum_{j=1}^{p+1}\frac{1}{\lambda_{j}}-\left(\sum_{j=1}^{p+1}\frac{{\left(\lambda_{j}+d\right)}^{2}}{\lambda_{j}{\left(\lambda_{j}+1\right)}^{2}}+{\left(d-1\right)}^{2}\sum_{j=1}^{p+1}\frac{\alpha_{j}^{2}}{{\left(\lambda_{j}+1\right)}^{2}}\right). \end{array}$$

It is clear that Δ=0 when *d* equals one, and then $\text{SMSE}\left({\hat{\beta }}_{{\text{MLE}}_{\left(\text{PMQL}\right)}}\right)=\text{SMSE}\left({\hat{\beta }}_{{\text{LE}}_{\left(\text{PMQL}\right)}}\right)$. Further, the estimator ${\hat{\beta }}_{{\text{LE}}_{\left(\text{PMQL}\right)}}$ is said to be superior to the estimator ${\hat{\beta }}_{{\text{MLE}}_{\left(\text{PMQL}\right)}}$ in the form of the SMSE criterion if and only if Δ > 0. Then, if we can find a *d*(0 < *d* < 1) such that Δ > 0, we can say that the estimator ${\hat{\beta }}_{{\text{LE}}_{\left(\text{PMQL}\right)}}$ is superior to ${\hat{\beta }}_{{\text{MLE}}_{\left(\text{PMQL}\right)}}$ in the PMQL regression model.

Kejian [22] showed that there exists a *d*(0 < *d* < 1) such that the Liu regression estimator has a lower SMSE than the ordinary least square estimator. Further, Kurtoğlu and Özkale [28] have proven that this property holds for the GLMs. The following two propositions show that this property holds in the PMQL Liu regression model.

**Proposition 1 .**

The total variance of the regression coefficient estimates of ${\hat{\beta }}_{{\text{LE}}_{\left(\text{PMQL}\right)}}$ (term I) and squared bias of ${\hat{\beta }}_{{\text{LE}}_{\left(\text{PMQL}\right)}}$ (term II) are continuous monotonically increasing and decreasing functions of *d*, respectively.

**Proof**

: The first derivative of term I in terms of *d* is

(26) $$\begin{array}{c} \frac{\partial \text{ term }I}{\partial d}=2\sum_{j=1}^{p+1}\frac{\left(\lambda_{j}+d\right)}{\lambda_{j}{\left(\lambda_{j}+1\right)}^{2}}, \end{array}$$

(27) $$\begin{array}{c} \underset{d⟶0^{+}}{\lim}\;\frac{\partial \text{ term }I}{\partial d}=2\sum_{j=1}^{p+1}\frac{1}{{\left(\lambda_{j}+1\right)}^{2}}, \end{array}$$

(28) $$\begin{array}{c} \underset{d⟶1^{-}}{\lim}\;\frac{\partial \text{ term }I}{\partial d}=2\sum_{j=1}^{p+1}\frac{1}{\lambda_{j}\left(\lambda_{j}+1\right)}. \end{array}$$

Since *λ*_*j*_ > 0 for all *j*, equation (26) is always positive for all *d*(0 < *d* < 1). Further, the derivative of the term I in the neighborhood of zero given in equation (27) and the derivative of the term I in the neighborhood of one given in equation (28) are positive.

The first derivative of term II in terms of *d* is

(29) $$\begin{array}{c} \frac{\partial \text{ term II}}{\partial d}=2\left(d-1\right)\sum_{j=1}^{p+1}\frac{\alpha_{j}^{2}}{{\left(\lambda_{j}+1\right)}^{2}}, \end{array}$$

(30) $$\begin{array}{c} \underset{d⟶0^{+}}{\lim}\frac{\partial \text{ term II}}{\partial d}=-2\sum_{j=1}^{p+1}\frac{\alpha_{j}^{2}}{{\left(\lambda_{j}+1\right)}^{2}}, \end{array}$$

(31) $$\begin{array}{c} \underset{d⟶1^{-}}{\lim}\frac{\partial \text{ term II}}{\partial d}=0. \end{array}$$

Since *λ*_*j*_ > 0 and *α*_*j*_^2^ > 0 for all *j*, equation (29) is always negative for all *d*(0 < *d* < 1). Further, the derivative of the term II in the neighborhood of zero given in equation (30) is negative, and the derivative of the term II in the neighborhood of one given in equation (31) is zero.

Then, it is shown that term I and term II are continuous monotonically increasing and decreasing functions of *d*, respectively.

**Proposition 2 .**

The SMSE $\left({\hat{\beta }}_{{\text{LE}}_{\left(\text{PMQL}\right)}}\right)$ given in equation (24) is a continuous monotonically increasing function of *d* when (*α*_max^2^_ − 1)/(1/*λ*_max_)+*α*_max^2^_ < *d* < 1, where *α*_max^2^_ is the maximum element of *α*_*j*_^2^(*j*=1,2,…, *p*+1) and *λ*_max_ is the maximum eigenvalue of the matrix $X^{\prime }\hat{W}X$.

**Proof**

The first derivative of equation (24) is

(32) $$\begin{array}{c} \frac{\partial \text{ SMSE}\left({\hat{\beta }}_{{\text{LE}}_{\left(\text{PMQL}\right)}}\right)}{\partial d}=2\sum_{j=1}^{p+1}\frac{\left(\lambda_{j}+d\right)}{\lambda_{j}{\left(\lambda_{j}+1\right)}^{2}}+2\left(d-1\right)\sum_{j=1}^{p+1}\frac{\alpha_{j}^{2}}{{\left(\lambda_{j}+1\right)}^{2}}. \end{array}$$

One can note that if the individual Liu parameter *d*_*j*_ > (*α*_*j*_^2^ − 1)/(1/*λ*_*j*_) + *α*_*j*_^2^,  ∀ *j* = 1,2,…, *p* + 1, equation (32) is positive. Then, it is clear that when (*α*_max_^2^ − 1)/(1/*λ*_max_) + *α*_max_^2^ < *d* < 1, equation (32) is always positive. Then, it is shown that the SMSE $\left({\hat{\beta }}_{{\text{LE}}_{\left(\text{PMQL}\right)}}\right)$ is a continuous monotonically increasing function of *d* when (*α*_max_^2^ − 1)/(1/*λ*_max_) + *α*_max_^2^ < *d* < 1.

Now, we can conclude that there is a possibility of finding a value *d*(0 < *d* < 1) based on the results of Proposition 1. Further, the results of Δ=0 given in equation (25) at *d* equal one, and Proposition 2 reveals that the Δ > 0 when (*α*_max_^2^ − 1)/(1/*λ*_max_)+*α*_max_^2^ < *d* < 1.

Then, it is shown that there exists a *d*(0 < *d* < 1) such that SMSE $\left({\hat{\beta }}_{{\text{LE}}_{\left(\text{PMQL}\right)}}\right)$ < SMSE $\left({\hat{\beta }}_{{\text{MLE}}_{\left(\text{PMQL}\right)}}\right)$.

The following theorem discusses the condition that the LE is superior to the MLE in the PMQL regression model.

**Theorem 1 .**

Let *λ*_*j*_(*λ*_*j*_+1)^2^ − (*λ*_*j*_+*d*)^2^ > 0,  (*j*=1,2 …, *p*+1) and $b_{d}=\text{Bias}\left({\hat{\beta }}_{{\text{LE}}_{\left(\text{PMQL}\right)}}\right)$. Then, $\text{MSE}\left({\hat{\beta }}_{{\text{MLE}}_{\left(\text{PMQL}\right)}}\right)-\text{MSE}\left({\hat{\beta }}_{{\text{LE}}_{\left(\text{PMQL}\right)}}\right)>0$ iff *b*_*d*_′(Γ(Λ^−1^ − (Λ+*I*)^−1^(Λ+*dI*)Λ^−1^(Λ+*dI*)(Λ+*I*)^−1^)Γ′)^−1^*b*_*d*_ < 1.

**Proof**

The difference between the MSE of the MLE and LE is derived as

(33) $$\begin{array}{c} \text{MSE}\left({\hat{\beta }}_{{\text{MLE}}_{\left(\text{PMQL}\right)}}\right)-\text{MSE}\left({\hat{\beta }}_{{\text{LE}}_{\left(\text{PMQL}\right)}}\right)\\ ={\left(X^{\prime }\hat{W}X\right)}^{-1}-{\left(X^{\prime }\hat{W}X+I\right)}^{-1}\left(X^{\prime }\hat{W}X+dI\right){\left(X^{\prime }\hat{W}X\right)}^{-1}\left(X^{\prime }\hat{W}X+dI\right){\left(X^{\prime }\hat{W}X+I\right)}^{-1}-b_{d}b_{d}^{\prime }. \end{array}$$

Now, we apply the spectral decomposition for the above matrix. Then, the difference can be written as

(34) $$\begin{array}{c} \text{MSE}\left({\hat{\beta }}_{{\text{MLE}}_{\left(\text{PMQL}\right)}}\right)-\text{MSE}\left({\hat{\beta }}_{{\text{LE}}_{\left(\text{PMQL}\right)}}\right)=\Gamma \Gamma^{\prime }{\left(X^{\prime }\hat{W}X\right)}^{-1}\Gamma \Gamma^{\prime }\\ -\Gamma \Gamma^{\prime }{\left(X^{\prime }\hat{W}X+I\right)}^{-1}\Gamma \Gamma^{\prime }\left(X^{\prime }\hat{W}X+dI\right)\Gamma \Gamma^{\prime }{\left(X^{\prime }\hat{W}X\right)}^{-1}\Gamma \Gamma^{\prime }\left(X^{\prime }\hat{W}X+dI\right)\Gamma \Gamma^{\prime }{\left(X^{\prime }\hat{W}X+I\right)}^{-1}\Gamma \Gamma^{\prime }-b_{d}b_{d}^{\prime }\\ =\Gamma \Lambda^{-1}\Gamma^{\prime }-\Gamma {\left(\Lambda +I\right)}^{-1}\left(\Lambda +dI\right)\Lambda^{-1}\left(\Lambda +dI\right){\left(\Lambda +I\right)}^{-1}\Gamma^{\prime }-b_{d}b_{d}^{\prime }\\ =\Gamma \left(\Lambda^{-1}-{\left(\Lambda +I\right)}^{-1}\left(\Lambda +dI\right)\Lambda^{-1}\left(\Lambda +dI\right){\left(\Lambda +I\right)}^{-1}\right)\Gamma^{\prime }-b_{d}b_{d}^{\prime }\\ =\Gamma \text{diag}{\left(\frac{1}{\lambda_{j}}-\frac{{\left(\lambda_{j}+d\right)}^{2}}{\lambda_{j}{\left(\lambda_{j}+1\right)}^{2}}\right)}_{j=1,2,\ldots ,p+1}\Gamma^{\prime }-b_{d}b_{d}^{\prime }. \end{array}$$

The diagonal matrix Λ^−1^ − (Λ+*I*)^−1^(Λ+*dI*)Λ^−1^(Λ+*dI*)(Λ+*I*)^−1^ is pd if *λ*_*j*_(*λ*_*j*_+1)^2^ − (*λ*_*j*_+*d*)^2^ > 0,  (*j*=1,2,…, *p*+1). Then, by Lemma A1 (Appendix 1), $\text{MSE}\left({\hat{\beta }}_{{\text{MLE}}_{\left(\text{PMQL}\right)}}\right)-\text{MSE}\left({\hat{\beta }}_{{\text{LE}}_{\left(\text{PMQL}\right)}}\right)>0$ if *b*_*d*_′(Γ(Λ^−1^ − (Λ+*I*)^−1^(Λ+*dI*)Λ^−1^(Λ+*dI*)(Λ+*I*)^−1^)Γ′)^−1^*b*_*d*_ < 1. It completes the proof.

The following theorem discusses the condition that the LE is superior to the RE in the PMQL regression model.

**Theorem 2 .**

Let *λ*_*j*_^2^(*λ*_*j*_+1)^2^ − (*λ*_*j*_+*d*)^2^(*λ*_*j*_+*k*)^2^ > 0,  (*j*=1,2,…, *p*+1) and $b_{k}=\text{Bias}\left({\hat{\beta }}_{{\text{RE}}_{\left(\text{PMQL}\right)}}\right)=-k{\left(X^{\prime }\hat{W}X+kI\right)}^{-1}$. Then, $\text{MSE}\left({\hat{\beta }}_{{\text{RE}}_{\left(\text{PMQL}\right)}}\right)-\text{MSE}\left({\hat{\beta }}_{{\text{LE}}_{\left(\text{PMQL}\right)}}\right)>0$ iff *b*_*d*_′(Γ((Λ+*kI*)^−1^Λ(Λ+*kI*)^−1^ − (Λ+*I*)^−1^(Λ+*dI*)Λ^−1^(Λ+*dI*)(Λ+*I*)^−1^)Γ′+*b*_*k*_*b*_*k*_′)^−1^*b*_*d*_ < 1.

**Proof**

: The difference between the MSE of the RE and LE is derived as

(35) $$\begin{array}{c} \text{MSE}\left({\hat{\beta }}_{{\text{RE}}_{\left(\text{PMQL}\right)}}\right)-\text{MSE}\left({\hat{\beta }}_{{\text{LE}}_{\left(\text{PMQL}\right)}}\right)\\ ={\left(X^{\prime }\hat{W}X+kI\right)}^{-1}\left(X^{\prime }\hat{W}X\right){\left(X^{\prime }\hat{W}X+kI\right)}^{-1}-\\ {\left(X^{\prime }\hat{W}X+I\right)}^{-1}\left(X^{\prime }\hat{W}X+dI\right){\left(X^{\prime }\hat{W}X\right)}^{-1}\left(X^{\prime }\hat{W}X+dI\right){\left(X^{\prime }\hat{W}X+I\right)}^{-1}+b_{k}b_{k}^{\prime }-b_{d}b_{d}^{\prime }, \end{array}$$

By applying the spectral decomposition for the above matrix, the difference can be written as

(36) $$\begin{array}{c} \text{MSE}\left({\hat{\beta }}_{{\text{RE}}_{\left(\text{PMQL}\right)}}\right)-\text{MSE}\left({\hat{\beta }}_{{\text{LE}}_{\left(\text{PMQL}\right)}}\right)=\Gamma \Gamma^{\prime }{\left(X^{\prime }\hat{W}X+kI\right)}^{-1}\Gamma \Gamma^{\prime }\left(X^{\prime }\hat{W}X\right)\Gamma \Gamma^{\prime }{\left(X^{\prime }\hat{W}X+kI\right)}^{-1}\Gamma \Gamma^{\prime }-\\ \Gamma \Gamma^{\prime }{\left(X^{\prime }\hat{W}X+I\right)}^{-1}\Gamma \Gamma^{\prime }\left(X^{\prime }\hat{W}X+dI\right)\Gamma \Gamma^{\prime }{\left(X^{\prime }\hat{W}X\right)}^{-1}\Gamma \Gamma^{\prime }\left(X^{\prime }\hat{W}X+dI\right)\Gamma \Gamma^{\prime }{\left(X^{\prime }\hat{W}X+I\right)}^{-1}\Gamma \Gamma^{\prime }+b_{k}b_{k}^{\prime }-b_{d}b_{d}^{\prime }\\ =\Gamma {\left(\Lambda +kI\right)}^{-1}\Lambda {\left(\Lambda +kI\right)}^{-1}\Gamma^{\prime }-\Gamma {\left(\Lambda +I\right)}^{-1}\left(\Lambda +dI\right)\Lambda^{-1}\left(\Lambda +dI\right){\left(\Lambda +I\right)}^{-1}\Gamma^{\prime }+b_{k}b_{k}^{\prime }-b_{d}b_{d}^{\prime }\\ =\Gamma \left({\left(\Lambda +kI\right)}^{-1}\Lambda {\left(\Lambda +kI\right)}^{-1}-{\left(\Lambda +I\right)}^{-1}\left(\Lambda +dI\right)\Lambda^{-1}\left(\Lambda +dI\right){\left(\Lambda +I\right)}^{-1}\right)\Gamma^{\prime }+b_{k}b_{k}^{\prime }-b_{d}b_{d}^{\prime }\\ =\Gamma \text{diag}{\left(\frac{\lambda_{j}}{{\left(\lambda_{j}+k\right)}^{2}}-\frac{{\left(\lambda_{j}+d\right)}^{2}}{\lambda_{j}{\left(\lambda_{j}+1\right)}^{2}}\right)}_{j=1,2,\ldots ,p+1}\Gamma^{\prime }+b_{k}b_{k}^{\prime }-b_{d}b_{d}^{\prime }\\ =\Gamma \text{diag}{\left(\frac{\lambda_{j}^{2}{\left(\lambda_{j}+1\right)}^{2}-{\left(\lambda_{j}+d\right)}^{2}{\left(\lambda_{j}+k\right)}^{2}}{\lambda_{j}{\left(\lambda_{j}+k\right)}^{2}{\left(\lambda_{j}+1\right)}^{2}}\right)}_{j=1,2,\ldots ,p+1}\Gamma^{\prime }+b_{k}b_{k}^{\prime }-b_{d}b_{d}^{\prime }. \end{array}$$

It is clear that *b*_*k*_*b*_*k*_′ is a pd matrix, and the diagonal matrix (Λ + *kI*)^−1^Λ(Λ + *kI*)^−1^ − (Λ + *I*)^−1^(Λ + *dI*)Λ^−1^(Λ + *dI*)(Λ + *I*)^−1^ is pd if *λ*_*j*_^2^(*λ*_*j*_ + 1)^2^ − (*λ*_*j*_ + *d*)^2^(*λ*_*j*_ + *k*)^2^ > 0,  (*j* = 1,2,…, *p* + 1).

Then, by Lemma A1 (Appendix 1), $\text{MSE}\left({\hat{\beta }}_{{\text{RE}}_{\left(\text{PMQL}\right)}}\right)-\text{MSE}\left({\hat{\beta }}_{{\text{LE}}_{\left(\text{PMQL}\right)}}\right)>0$ if *b*_*d*_′(Γ((Λ+*kI*)^−1^Λ(Λ+*kI*)^−1^ − (Λ+*I*)^−1^(Λ+*dI*)Λ^−1^(Λ+*dI*)(Λ+*I*)^−1^)Γ′+*b*_*k*_*b*_*k*_′)^−1^*b*_*d*_ < 1. It completes the proof.

### 3.2. Estimation of the Liu Parameter *d*

Based on the MSE properties of the PMQL Liu regression estimator discussed in Section 3.1, it is clear that the performance of ${\hat{\beta }}_{{\text{LE}}_{\left(\text{PMQL}\right)}}$ depends on the optimum value of the Liu parameter *d*. The optimal value of any individual Liu parameter *d*_*j*_ can be found by setting equation (32) to zero and solving for *d*_*j*_. Then, it is obtained as

(37) $$\begin{array}{c} d_{j}=\frac{\alpha_{j}^{2}-1}{\left(1/\lambda_{j}\right)+\alpha_{j}^{2}},\;j=1,2,\ldots ,p+1. \end{array}$$

From equation (37), we can note that the optimum value is negative when *α*_*j*_^2^ < 1 (*j*=1,2,…, *p*+1) and otherwise positive. Since the value of the *d* is limited between 0 and 1, we should use the “max” operator to ensure the estimated value of the *d* is nonnegative.

In this subsection, we adopt some notable existing Liu parameter estimators in order to estimate the Liu parameter in ${\hat{\beta }}_{{\text{LE}}_{\left(\text{PMQL}\right)}}$. They are summarized in Table 1 (Appendix 2). We define the Liu parameter estimators ${\hat{D}}_{1},{\hat{D}}_{2}-{\hat{D}}_{3},$ and ${\hat{D}}_{4}-{\hat{D}}_{5}$ for ${\hat{\beta }}_{{\text{LE}}_{\left(\text{PMQL}\right)}}$ based on the theoretical works of Hoerl and Kennard [20], Kibria [35], and Khalaf and Shukur [36], respectively.

To estimate the ridge parameter in the PMQL ridge regression estimator, Tharshan and Wijekoon [19] discussed 12 various estimation methods. Among all, they recommended three ridge parameter estimators, ${\hat{k}}_{2},{\hat{k}}_{7}$, and ${\hat{k}}_{12}$, based on the works of Hoerl and Kennard [20], Nomura [37], and Muniz and Kibria [38]. These ridge parameter estimators are summarized in Table 1 (Appendix 2). Therefore, ${\hat{k}}_{2},{\hat{k}}_{7}$, and ${\hat{k}}_{12}$ will be utilized to estimate *k* in ${\hat{\beta }}_{{\text{RE}}_{\left(PMQL\right)}}$ in the simulation study.

## 4. The Monte Carlo Simulation Study

In this section, a simulation study is carried out to evaluate the performance of the MLE_(PMQL)_, PMQL ridge regression estimators, PMQL Liu regression estimators based on the various ridge, and Liu parameter estimators, respectively, as discussed in Section 3.2. We compare the performance of different estimators in the SMSE sense. A brief discussion about the simulation study is given in the following.

### 4.1. The Design of the Simulation Study

Since the degrees of the correlation (*ρ*) between the covariates greatly depends on the performance of the various estimators, we generate the covariates with several degrees of multicollinearity by following the same formula as used by McDonald and Galarneau [39]. The formula is given as follows:

(38) $$\begin{array}{c} x_{ij}=\sqrt{\left(1-\rho^{2}\right)}m_{ij}+\rho m_{i,p+1},\;i=1,2,\ldots ,n,\;j=1,2,\ldots ,p, \end{array}$$

where *m*_*ij*_'s are independent standard normal pseudorandom numbers. The response variable *y* of the PMQL regression model is generated from the PMQL (*μ*_*i*_, *α*, *δ*) by using the inverse transform method, where *μ*_*i*_=exp(*x*_*i*_′*β*),  *i*=1,2,…, *n*. The starting values of the slope parameters are selected such that ∑_*j*=1_^*p*^*β*_*j*_^2^=1 and *β*_1_=*β*_2_=⋯=*β*_*p*_.

Table 2 summarizes the factors and their levels that are considered in this design. Since either higher increments or decrements of variation of *y* may lead to a negative impact on the performance of estimators [19, 24, 40, 41], we vary *β*_0_, *α*, and *δ*. When we decrease the value of *β*_0_, the average values of the *y*_*i*_(*μ*_*i*_),  *i*=1,2,…, *n* will decrease. This phenomenon leads to having more zeros of *y*, which makes very less variation in the sample. Further, from Figure 1, we can observe that changing the value of the overdispersion parameters *α* or *δ* affects the variation of *y*.

The simulation is repeated 1000 times. To judge the performance of the different estimators, we obtain the SMSE values of different estimators by using the following equation:

(39) $$\begin{array}{c} \hat{\text{SMSE}}\left(\hat{\beta }\right)=\frac{\sum_{r=1}^{1000}{\left({\hat{\beta }}_{r}-\beta \right)}^{\prime }\left({\hat{\beta }}_{r}-\beta \right)}{1000}, \end{array}$$

where ${\hat{\beta }}_{r}$ is an estimator of *β* at the *r*^th^ replication.

### 4.2. Results and Discussion of the Simulation Study

The estimated SMSE values of the Monte Carlo Simulations are summarized in Tables 3–10 (Appendix 2) for the selected situations shown in Table 2. The minimum SMSE in each combination of different factor levels is presented in bold. In general, we can note that the LE_(PMQL)_ is more efficient than the MLE_(PMQL)_ and RE_(PMQL)_ in all cases reviewed in this study. Further, the performances of all regression coefficient estimators are affected by the factors of degrees of the correlation among the covariates, the sample size, the value of the intercept, the number of covariates, and the values of the overdispersion parameters.

It can be noticed that as the degrees of correlation increase, the estimated SMSE of the MLE_(PMQL)_ increases, and the PMQL Liu regression estimators having the estimated *d* values *D*_1_, *D*_2_, *D*_3_, and *D*_4_ are also affected negatively in some cases. However, the PMQL Liu regression estimator based on the *D*_5_ estimator does not affect and yields consistently a smaller SMSE in all cases. Further, its estimated SMSE decreases with *ρ*.

When the sample size increases, SMSE of the MLE_(PMQL)_ and the PMQL ridge and Liu regression estimators decrease. This phenomenon reveals that the asymptotic property holds for all given estimators. Further, in a given sample size, the PMQL Liu regression estimator based on the *D*_5_ estimator performs better than the MLE_(PMQL)_ and PMQL ridge regression estimators for all given situations.

It is clearly observed that as the *β*_0_ decreases from 1 to −1, the SMSE of the MLE_(PMQL)_ and PMQL Liu regression estimators based on the *D*_1_, *D*_3_, and *D*_4_ estimators are increased with a higher amount and based on *D*_2_ estimator are also affected negatively for some cases. However, this change does not affect the performance of the PMQL Liu regression estimator based on the *D*_5_ estimator basically.

The increasing number of covariates affects the performance of the MLE_(PMQL)_ negatively in all given situations, and the PMQL ridge regression estimators also affect negatively in some given situations. However, the PMQL Liu regression estimator based on *D*_5_ estimator does not affect when increasing the number of covariates in all given situations. Further, when *p* increases for a given combination of *ρ*, *n*, *β*_0_, *α*, *δ*, the SMSEs produced by PMQL Liu regression estimator based on *D*_5_ estimator decrease.

We can clearly note that the increment of either the overdispersion parameter *α* (0.03 to 0.25) or *δ* (0.02 to 0.04) shows a positive impact on the performance of different estimators. From Figure 1, we can note that, in both situations, the variance of *y* decreases. These results are in line with the simulated results of [17, 41].

Then, based on the simulation study results, we may say that the Liu parameter estimator *D*_5_ is the best option to estimate the Liu parameter *d* in the ${\hat{\beta }}_{{\text{LE}}_{\left(\text{PMQL}\right)}}$.

## 5. Applications

In this section, we use a simulated data set to show the applicability of the PMQL Liu regression model for the count data set with a higher index of dispersion and long right tail over the existing Liu regression models for count data, namely, NB Liu regression and Poisson Liu regression models. Further, we illustrate the applicability of the PMQL Liu regression estimator over the MLE_(PMQL)_ by using a real data set also. We have observed that the PMQL Liu regression estimator based on *D*_5_ estimator performs well in the simulation study. Further, the same Liu parameter estimator was recommended by Månsson [25] for the NB Liu regression model and by Månsson [23] for the Poisson Liu regression model. Then, we will use the *D*_5_ estimator to estimate the Liu parameter in all considered different Liu regression estimators.

### 5.1. Simulated Data Application

**Figure 2:**
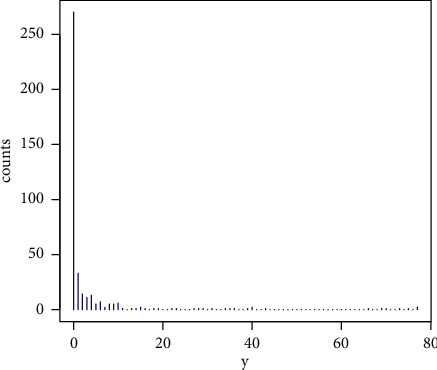
The distribution of the response variable *y*.

A data set with *p*=4,  *ρ*=0.9999, and *n*=400 was simulated by using the method discussed in Section 4.1 in order to show the applicability of the proposed Liu regression model over the NB Liu and Poisson Liu regression models. The skewness, excess kurtosis, and index of dispersion of the response variable *y* are 4.628, 20.113, and 35.217, respectively. Then, the distribution of *y* has higher positive skewness, a long right tail, and higher overdispersion (index of dispersion >1).  Figure 2 also illustrates the distribution of the response variable *y*.  Table 11 displays the estimated regression coefficients, their standard errors (SEs) (in parentheses), SMSE values, and Akaike information criterion (AIC) values for the given regression models. We can clearly observe that the PMQL Liu regression model produces smaller SEs for the coefficients of covariates, SMSE value, and AIC value than the other regression models. Then, we may say that the PMQL Liu regression model gives a better performance than the other regression models based on the Liu estimator.

### 5.2. Real Data Application

The applicability of the PMQL Liu estimator over the MLE is illustrated by using the Swedish football data set, which consists of the Swedish football teams' performance in the top Swedish league (Allsvenskan) for the year 2012. Qasim et al. [32] used a similar data set during the year 2018 to fit a Poisson regression model. It contains 242 observations and represents the number of full-time home team goals (*y*), the pinnacle home win odds (*x*_1_), the pinnacle away win odds (*x*_2_), the maximum odds portal home win (*x*_3_), the maximum odds portal away win (*x*_4_), the average odds portal home win (*x*_5_), and the average odds portal away win (*x*_6_).

The conditional number, which is the ratio of the maximum to minimum eigenvalues, is 33460.350, which is clearly much larger than 1000. The index of dispersion of *y* is 1.201, which is greater than one. These results indicate that there exists severe multicollinearity among the covariates, and *y* is overdispersed. Further, to examine whether the PMQL distribution is suitable for *y*, the Chi-square (*χ*^2^) goodness of fit test is employed. *χ*^2^ value is computed as 3.159 with a *p*-value equal to 0.531. Then, this test confirms that the PMQL distribution fits well for this response variable *y*.

Table 12 lists the estimated regression coefficients, their standard errors (SEs) (in parentheses), and SMSE values for the MLE_(PMQL)_ and PMQL Liu estimator. It can be clearly noted that the PMQL Liu estimator produces smaller SEs and SMSE than the MLE_(PMQL)_. Then, we can conclude that the PMQL Liu estimator performs better than MLE_(PMQL)_ for this real data set that has severe multicollinearity issues.

Now, we justify Theorem 1 by using the real-world application. The necessary conditions in Theorem 1 are holding asmin(*λ*_*j*_(*λ*_*j*_+1)^2^ − (*λ*_*j*_+*d*)^2^)_*j*=1_^*p*+1^=89.004 > 0, the minimum eigenvalue of the difference matrix

(40) $$\begin{array}{c} \text{MSE}\left({\hat{\beta }}_{{\text{MLE}}_{\left(\text{PMQL}\right)}}\right)-\text{MSE}\left({\hat{\beta }}_{{\text{LE}}_{\left(\text{PMQL}\right)}}\right)\text{equals }7.134e^{-13}>0\\ b_{d}^{\prime }{\left(\Gamma \left(\left(\Lambda^{-1}-{\left(\Lambda +I\right)}^{-1}\left(\Lambda +dI\right)\Lambda^{-1}\left(\Lambda +dI\right){\left(\Lambda +I\right)}^{-1}\right)\right)\Gamma^{\prime }\right)}^{-1}b_{d}=0.501<1, \end{array}$$

and hence Theorem 1 is justified.

## 6. Conclusion

This paper introduced the Liu estimator for the Poisson-Modification of Quasi Lindley (PMQL) regression model instead of the maximum likelihood estimator (MLE) in order to mitigate the multicollinearity and overdispersion issues. A comprehensive Monte Carlo simulation study was conducted to compare the performance of the MLE, the PMQL Liu regression estimator, and the existing biased estimator, namely, the PMQL ridge regression estimator. The scalar mean square error (SMSE) was considered as the evaluation criterion. The simulation study results revealed that the performance of the different estimators is affected by the different levels of factors such as correlations between the covariates, sample size, number of covariates, intercept, and the overdispersion parameters of the PMQL regression model. Further, the PMQL Liu regression estimator based on the Liu parameter estimator *D*_5_ shows a better performance than the other estimators in all situations reviewed in the simulation study. The results of a simulated data set show that the PMQL Liu regression model performs better than the existing count Liu regression models, namely, the negative binomial Liu regression and the Poisson Liu regression models, by mitigating higher overdispersion and multicollinearity. Further, a real-world application also shows that the PMQL Liu regression estimator based on the Liu parameter estimator *D*_5_ has a better performance than the MLE. Therefore, based on the simulation study and applications, the PMQL Liu regression estimator based on the Liu parameter estimator *D*_5_ is recommended for analyzing the overdispersed count responses with intercorrelated covariates.

## Figures and Tables

**Table 1:** The Liu and ridge parameter estimators for the PMQL Liu and ridge regression models.

Author(s)	Liu and ridge parameter estimators
Hoerl and Kennard [20]	${\hat{D}}_{1}=\max\left(0,{\hat{\alpha }}_{\max}^{2}-1/\left(1/{\hat{\lambda }}_{\max}\right)+{\hat{\alpha }}_{\max}^{2}\right)$
Kibria [35]	${\hat{D}}_{2}=\max\left(0,\text{median}\left({\hat{\alpha }}_{j}^{2}-1/\left(1/{\hat{\lambda }}_{j}\right)+{\hat{\alpha }}_{j}^{2}\right)\right)$
	${\hat{D}}_{3}=\max\left(0,\sum_{j=1}^{p+1}\left({\hat{\alpha }}_{j}^{2}-1/\left(1/{\hat{\lambda }}_{j}\right)+{\hat{\alpha }}_{j}^{2}\right)/\left(p+1\right)\right)$
Khalaf and Shukur [36]	${\hat{D}}_{4}=\max\left(0,\max\left({\hat{\alpha }}_{j}^{2}-1/\left(1/{\hat{\lambda }}_{j}\right)+{\hat{\alpha }}_{j}^{2}\right)\right)$
	${\hat{D}}_{5}=\max\left(0,\min\left({\hat{\alpha }}_{j}^{2}-1/\left(1/{\hat{\lambda }}_{j}\right)+{\hat{\alpha }}_{j}^{2}\right)\right)$
Nomura [37]	${\hat{k}}_{2}=p+1/\sum_{j=1}^{p+1}\left({\hat{\alpha }}_{j}^{2}/\left(1+\left(1+\lambda_{j}{\left({\hat{\alpha }}_{j}^{2}\right)}^{1/2}\right)\right)\right)$ .
Muniz and Kibria [38]	${\hat{k}}_{7}=\max\left(1/\sqrt{1/{\hat{\alpha }}_{j}^{2}}\right),\;j=1,2,\ldots ,p+1$ ,
	${\hat{k}}_{12}=\text{median}\left(\sqrt{1/{\hat{\alpha }}_{j}^{2}}\right),\;j=1,2,\ldots p+1$ ,

**Table 2:** Factors and their levels that are considered in the design of the simulation study.

Factors	Levels	Purpose
Degrees of correlation (*ρ*)	0.99, 0.999, and 0.9999	To examine the performance of different estimators when increasing *ρ*.
Sample size (*n*)	10, 20, 30, 40, and 100	To examine the asymptotic property and the performance of different estimators with *n*.
Number of covariates (*ρ*)	2 and 4	To examine the performance of the various estimators when increasing *p*.
		To examine the performance of the various estimators when changing the variation of *y*:
Intercept (*β*_0_)	−1 and 1	(i) By changing *β*_0_.
Overdispersion parameter 1 (*α*)	0.03 and 0.25	(ii) By changing *α*.
Overdispersion parameter 2 (*δ*)	0.02 and 0.04	(iii) By changing *δ*.

**Table 3:** Estimated SMSE values for different *n*, *ρ*, *k*, *d*,  and *β*_0_ when *δ*=0.02,  *α*=0.03,  and *p*=2.

*ρ*=0.99										
Estimator	*β*_0_=−1					*β*_0_=1				
	*n*=10	*n*=20	*n*=30	*n*=40	*n*=100	*n*=10	*n*=20	*n*=30	*n*=40	*n*=100
MLE	452.489	174.282	108.926	78.421	29.614	414.628	159.988	99.952	71.908	27.170
RE (*k*_2_)	1.589	1.349	1.189	1.080	0.768	1.856	1.758	1.686	1.635	1.475
RE (*k*_7_)	1.658	1.412	1.227	1.087	0.641	1.571	1.298	1.110	0.975	0.583
RE (*k*_12_)	1.709	1.477	1.298	1.145	0.667	1.759	1.630	1.545	1.493	1.271
LE (*D*_1_)	86.420	53.340	41.202	33.485	16.218	**1.567**	**1.295**	**1.108**	**0.974**	**0.582**
LE (*D*_2_)	3.646	3.137	2.494	2.083	0.795	**1.567**	**1.295**	**1.108**	**0.974**	**0.582**
LE (*D*_3_)	5.060	3.487	2.749	2.250	1.010	**1.567**	**1.295**	**1.108**	**0.974**	**0.582**
LE (*D*_4_)	49.373	37.725	31.882	27.274	14.777	**1.567**	**1.295**	**1.108**	**0.974**	**0.582**
LE (*D*_5_)	**1.564**	**1.297**	**1.116**	**0.986**	**0.605**	**1.567**	**1.295**	**1.108**	**0.974**	**0.582**

*ρ*=0.999										
MLE	4396.674	1692.037	1056.591	760.296	286.932	4029.486	1554.024	970.048	697.530	263.417
RE (*k*_2_)	1.586	1.344	1.184	1.074	0.763	1.855	1.757	1.686	1.634	1.475
RE (*k*_7_)	1.656	1.408	1.222	1.082	0.632	1.565	1.288	1.097	0.959	0.548
RE (*k*_12_)	1.711	1.474	1.291	1.132	0.646	1.760	1.631	1.548	1.497	1.277
LE (*D*_1_)	823.212	507.768	384.280	314.227	151.077	**1.561**	**1.286**	**1.095**	**0.958**	**0.548**
LE (*D*_2_)	22.182	17.975	14.279	11.114	2.237	**1.561**	**1.286**	**1.095**	**0.958**	**0.548**
LE (*D*_3_)	35.171	22.122	16.545	12.810	4.197	**1.561**	**1.286**	**1.095**	**0.958**	**0.548**
LE (*D*_4_)	463.151	353.349	297.506	254.383	136.779	**1.561**	**1.286**	**1.095**	**0.958**	**0.548**
LE (*D*_5_)	**1.559**	**1.289**	**1.104**	**0.972**	**0.574**	**1.561**	**1.286**	**1.095**	**0.958**	**0.548**

*ρ*=0.9999										
MLE	43836.060	16872.260	10534.020	7579.503	2860.172	40177.070	15496.860	9671.687	6954.122	2625.934
RE (*k*_2_)	1.585	1.344	1.183	1.074	0.762	1.855	1.757	1.686	1.634	1.475
RE (*k*_7_)	1.656	1.408	1.222	1.081	0.631	1.565	1.287	1.095	0.957	0.546
RE (*k*_12_)	1.711	1.474	1.290	1.131	0.644	1.760	1.631	1.548	1.498	1.277
LE (*D*_1_)	8201.712	4973.109	3898.417	3163.635	1503.131	**1.561**	**1.285**	**1.094**	**0.956**	**0.545**
LE (*D*_2_)	208.113	165.643	132.270	101.672	17.001	**1.561**	**1.285**	**1.094**	**0.956**	**0.545**
LE (*D*_3_)	336.983	208.402	154.438	118.589	36.359	**1.561**	**1.285**	**1.094**	**0.956**	**0.545**
LE (*D*_4_)	4596.616	3511.205	2954.507	2525.475	1356.645	**1.561**	**1.285**	**1.094**	**0.956**	**0.545**
LE (*D*_5_)	**1.559**	**1.288**	**1.103**	**0.970**	**0.571**	**1.561**	**1.285**	**1.094**	**0.956**	**0.545**

*Note*. RE (*a*) indicates the ridge estimator based on the ridge parameter estimator a, LE (*b*) indicates the Liu estimator based on the Liu parameter estimator *b*, and bold values indicate the minimum SMSEs.

**Table 4:** Estimated SMSE values for different *n*, *ρ*, *k*, *d*,  and *β*_0_ when *δ*=0.04,  *α*=0.03,  and *p*=2.

*ρ*=0.99										
Estimator	*β*_0_=−1					*β*_0_=1				
	*n*=10	*n*=20	*n*=30	*n*=40	*n*=100	*n*=10	*n*=20	*n*=30	*n*=40	*n*=100
MLE	245.039	93.937	58.668	42.234	15.927	210.562	81.256	50.776	36.539	13.806
RE (*k*_2_)	1.362	1.084	0.927	0.831	0.595	1.760	1.631	1.551	1.335	1.368
RE (*k*_7_)	1.433	1.109	0.902	0.763	0.450	1.321	0.992	0.802	0.680	0.393
RE (*k*_12_)	1.529	1.254	1.074	0.944	0.529	1.577	1.380	1.267	1.199	0.928
LE (*D*_1_)	70.111	37.569	27.385	21.437	9.560	**1.315**	**0.988**	**0.799**	**0.677**	**0.391**
LE (*D*_2_)	3.014	1.915	1.413	1.064	0.455	**1.315**	**0.988**	**0.799**	**0.677**	**0.391**
LE (*D*_3_)	3.980	2.187	1.530	1.155	0.480	**1.315**	**0.988**	**0.799**	**0.677**	**0.391**
LE (*D*_4_)	48.183	31.266	24.062	19.472	9.242	**1.315**	**0.988**	**0.799**	**0.677**	**0.391**
LE (*D*_5_)	**1.318**	**1.006**	**0.826**	**0.710**	**0.423**	**1.315**	**0.988**	**0.799**	**0.677**	**0.391**

*ρ*=0.999										
MLE	2380.463	911.567	568.777	409.234	154.218	2046.259	789.199	492.750	354.411	133.844
RE (*k*_2_)	1.357	1.078	0.921	0.825	0.591	1.759	1.630	1.550	1.500	1.369
RE (*k*_7_)	1.430	1.104	0.895	0.755	0.387	1.311	0.975	0.779	0.651	0.333
RE (*k*_12_)	1.537	1.255	1.072	0.936	0.516	1.577	1.382	1.270	1.205	0.933
LE (*D*_1_)	668.982	360.132	260.603	202.710	88.570	**1.306**	**0.972**	**0.777**	**0.650**	**0.332**
LE (*D*_2_)	18.050	9.434	6.071	4.062	0.620	**1.306**	**0.972**	**0.777**	**0.650**	**0.332**
LE (*D*_3_)	26.753	12.123	7.442	4.843	0.868	**1.306**	**0.972**	**0.777**	**0.650**	**0.332**
LE (*D*_4_)	454.645	293.149	224.734	181.166	84.798	**1.306**	**0.972**	**0.777**	**0.650**	**0.332**
LE (*D*_5_)	**1.310**	**0.992**	**0.807**	**0.686**	**0.371**	**1.306**	**0.972**	**0.777**	**0.650**	**0.332**

*ρ*=0.9999										
MLE	23733.270	9089.415	5670.356	4079.535	1537.169	20402.520	7869.867	4912.825	3533.315	1334.242
RE (*k*_2_)	1.357	1.077	0.921	0.825	0.591	1.759	1.630	1.550	1.500	1.369
RE (*k*_7_)	1.430	1.103	0.895	0.754	0.386	1.310	0.973	0.777	0.648	0.326
RE (*k*_12_)	1.538	1.256	1.073	0.935	0.514	1.577	1.383	1.270	1.206	0.935
LE (*D*_1_)	6686.193	3595.616	2566.089	2026.436	880.321	**1.305**	**0.971**	**0.775**	**0.647**	**0.325**
LE (*D*_2_)	169.962	83.376	52.921	35.078	2.784	**1.305**	**0.971**	**0.775**	**0.647**	**0.325**
LE (*D*_3_)	254.364	111.777	66.952	42.161	5.245	**1.305**	**0.971**	**0.775**	**0.647**	**0.325**
LE (*D*_4_)	4518.941	2913.413	2231.176	1797.415	840.163	**1.305**	**0.971**	**0.775**	**0.647**	**0.325**
LE (*D*_5_)	**1.309**	**0.990**	**0.805**	**0.684**	**0.365**	**1.305**	**0.971**	**0.775**	**0.647**	**0.325**

*Note*. RE (*a*) indicates the ridge estimator based on the ridge parameter estimator a, LE (*b*) indicates the Liu estimator based on the Liu parameter estimator *b*, and bold values indicate the minimum SMSEs.

**Table 5:** Estimated SMSE values for different *n*, *ρ*, *k*, *d*,  and *β*_0_ when *δ*=0.02,  *α*=0.25,  and *p*=2.

*ρ*=0.99										
Estimator	*β*_0_=−1					*β*_0_=1				
	*n*=10	*n*=20	*n*=30	*n*=40	*n*=100	*n*=10	*n*=20	*n*=30	*n*=40	*n*=100
MLE	372.624	143.374	89.597	64.506	24.352	335.750	129.559	80.947	58.240	22.006
RE (*k*_2_)	1.524	1.268	1.106	0.999	0.709	1.830	1.721	1.646	1.593	1.440
RE (*k*_7_)	1.596	1.323	1.127	0.984	0.556	1.500	1.205	1.012	0.879	0.513
RE (*k*_12_)	1.657	1.408	1.225	1.071	0.611	1.712	1.562	1.468	1.410	1.171
LE (*D*_1_)	81.482	48.077	36.358	29.354	13.841	**1.495**	**1.202**	**1.010**	**0.877**	**0.512**
LE (*D*_2_)	3.476	2.801	2.138	1.738	0.655	**1.495**	**1.202**	**1.010**	**0.877**	**0.512**
LE (*D*_3_)	4.793	3.092	2.332	1.860	0.783	**1.495**	**1.202**	**1.010**	**0.877**	**0.512**
LE (*D*_4_)	49.919	36.118	29.627	24.850	12.838	**1.495**	**1.202**	**1.010**	**0.877**	**0.512**
LE (*D*_5_)	**1.494**	**1.207**	**1.023**	**0.895**	**0.539**	**1.495**	**1.202**	**1.010**	**0.877**	**0.512**

*ρ*=0.999										
MLE	3620.475	1391.796	1056.591	625.309	235.911	3262.905	1258.426	785.586	564.931	213.346
RE (*k*_2_)	1.520	1.263	1.184	0.993	0.704	1.829	1.720	1.645	1.593	1.441
RE (*k*_7_)	1.594	1.319	1.222	0.978	0.546	1.493	1.194	0.997	0.859	0.473
RE (*k*_12_)	1.661	1.406	1.291	1.061	0.589	1.712	1.563	1.471	1.415	1.177
LE (*D*_1_)	787.324	458.141	384.280	276.544	128.660	**1.489**	**1.191**	**0.995**	**0.858**	**0.472**
LE (*D*_2_)	21.560	15.514	14.279	8.557	1.508	**1.489**	**1.191**	**0.995**	**0.858**	**0.472**
LE (*D*_3_)	33.215	18.962	16.545	9.874	2.662	**1.489**	**1.191**	**0.995**	**0.858**	**0.472**
LE (*D*_4_)	468.722	339.052	297.506	231.827	118.588	**1.489**	**1.191**	**0.995**	**0.858**	**0.472**
LE (*D*_5_)	**1.488**	**1.197**	**1.104**	**0.878**	**0.502**	**1.489**	**1.191**	**0.995**	**0.858**	**0.472**

*ρ*=0.9999										
MLE	36096.870	13878.260	8663.555	6233.728	2351.556	32533.570	12549.090	7832.527	5632.137	2126.778
RE (*k*_2_)	1.520	1.262	1.100	0.993	0.704	1.829	1.720	1.645	1.593	1.441
RE (*k*_7_)	1.594	1.319	1.121	0.978	0.545	1.493	1.192	0.995	0.857	0.469
RE (*k*_12_)	1.662	1.405	1.218	1.060	0.586	1.712	1.564	1.471	1.416	1.177
LE (*D*_1_)	7842.881	4570.904	3415.339	2780.195	1273.319	**1.489**	**1.190**	**0.993**	**0.856**	**0.468**
LE (*D*_2_)	200.651	143.093	106.295	77.311	10.414	**1.489**	**1.190**	**0.993**	**0.856**	**0.468**
LE (*D*_3_)	317.588	177.880	124.068	90.144	21.795	**1.489**	**1.190**	**0.993**	**0.856**	**0.468**
LE (*D*_4_)	4658.671	3368.218	2747.316	2301.456	1175.937	**1.489**	**1.190**	**0.993**	**0.856**	**0.468**
LE (*D*_5_)	**1.487**	**1.196**	**1.008**	**0.876**	**0.498**	**1.489**	**1.190**	**0.993**	**0.856**	**0.468**

*Note*. RE (*a*) indicates the ridge estimator based on the ridge parameter estimator a, LE (*b*) indicates the Liu estimator based on the Liu parameter estimator *b*, and bold values indicate the minimum SMSEs.

**Table 6:** Estimated SMSE values for different *n*, *ρ*, *k*, *d*,  and *β*_0_ when *δ*=0.04,  *α*=0.25,  and *p*=2.

*ρ*=0.99										
Estimator	*β*_0_=−1					*β*_0_=1				
	*n*=10	*n*=20	*n*=30	*n*=40	*n*=100	*n*=10	*n*=20	*n*=30	*n*=40	*n*=100
MLE	227.529	87.144	54.416	39.171	14.768	193.512	74.676	46.666	33.583	12.689
RE (*k*_2_)	1.330	1.050	0.896	0.802	0.577	1.746	1.613	1.533	1.483	1.355
RE (*k*_7_)	1.400	1.068	0.862	0.725	0.416	1.285	0.954	0.766	0.648	0.376
RE (*k*_12_)	1.503	1.228	1.052	0.924	0.521	1.548	1.343	1.226	1.157	0.882
LE (*D*_1_)	67.852	36.245	25.990	20.327	8.977	**1.279**	**0.949**	**0.763**	**0.645**	**0.374**
LE (*D*_2_)	2.899	1.772	1.305	0.976	0.430	**1.279**	**0.949**	**0.763**	**0.645**	**0.374**
LE (*D*_3_)	3.811	2.033	1.413	1.052	0.448	**1.279**	**0.949**	**0.763**	**0.645**	**0.374**
LE (*D*_4_)	47.518	30.250	23.033	18.542	8.692	**1.279**	**0.949**	**0.763**	**0.645**	**0.374**
LE (*D*_5_)	**1.284**	**0.970**	**0.793**	**0.680**	**0.407**	**1.279**	**0.949**	**0.763**	**0.645**	**0.374**

*ρ*=0.999										
MLE	2210.274	845.575	527.502	379.519	142.979	1880.557	725.279	452.854	325.727	123.011
RE (*k*_2_)	1.326	1.044	0.890	0.797	0.573	1.745	1.612	1.533	1.483	1.356
RE (*k*_7_)	1.397	1.063	0.855	0.717	0.363	1.275	0.935	0.742	0.617	0.312
RE (*k*_12_)	1.512	1.228	1.052	0.916	0.505	1.548	1.345	1.230	1.164	0.887
LE (*D*_1_)	648.258	337.046	243.617	191.393	83.591	**1.270**	**0.933**	**0.740**	**0.616**	**0.311**
LE (*D*_2_)	17.334	8.451	5.202	3.470	0.535	**1.270**	**0.933**	**0.740**	**0.616**	**0.311**
LE (*D*_3_)	25.449	11.022	6.590	4.168	0.712	**1.270**	**0.933**	**0.740**	**0.616**	**0.311**
LE (*D*_4_)	448.155	283.402	215.143	172.414	79.621	**1.270**	**0.933**	**0.740**	**0.616**	**0.311**
LE (*D*_5_)	**1.276**	**0.955**	**0.773**	**0.655**	**0.351**	**1.270**	**0.933**	**0.740**	**0.616**	**0.311**

*ρ*=0.9999										
MLE	22036.400	8431.337	5258.827	3783.276	1425.136	18750.330	7232.441	4515.045	3247.348	1226.248
RE (*k*_2_)	1.325	1.044	0.890	0.796	0.573	1.745	1.612	1.533	1.483	1.356
RE (*k*_7_)	1.396	1.062	0.854	0.716	0.361	1.274	0.934	0.739	0.613	0.305
RE (*k*_12_)	1.514	1.230	1.052	0.915	0.503	1.548	1.346	1.230	1.165	0.889
LE (*D*_1_)	6435.778	3365.452	2444.748	1882.106	822.170	**1.269**	**0.931**	**0.737**	**0.612**	**0.304**
LE (*D*_2_)	161.526	74.435	44.844	29.430	2.132	**1.269**	**0.931**	**0.737**	**0.612**	**0.304**
LE (*D*_3_)	241.493	101.296	58.745	35.786	3.880	**1.269**	**0.931**	**0.737**	**0.612**	**0.304**
LE (*D*_4_)	4456.390	2815.842	2135.848	1710.447	788.719	**1.269**	**0.931**	**0.737**	**0.612**	**0.304**
LE (*D*_5_)	**1.275**	**0.954**	**0.771**	**0.652**	**0.344**	**1.269**	**0.931**	**0.737**	**0.612**	**0.304**

*Note*. RE (*a*) indicates the ridge estimator based on the ridge parameter estimator a, LE (*b*) indicates the Liu estimator based on the Liu parameter estimator *b*, and bold values indicate the minimum SMSEs.

**Table 7:** Estimated SMSE values for different *n*, *ρ*, *k*, *d*,  and *β*_0_ when *δ*=0.02,  *α*=0.03,  and *p*=4.

*ρ*=0.99										
Estimator	*β*_0_=−1					*β*_0_=1				
	*n*=10	*n*=20	*n*=30	*n*=40	*n*=100	*n*=10	*n*=20	*n*=30	*n*=40	*n*=100
MLE	2300.301	639.879	369.034	259.458	93.662	1987.509	563.548	326.892	230.407	83.557
RE (*k*_2_)	1.512	1.257	1.100	0.994	0.720	1.850	1.766	1.713	1.677	1.584
RE (*k*_7_)	1.537	1.252	1.060	0.925	0.589	1.441	1.153	0.977	0.860	0.566
RE (*k*_12_)	1.809	1.736	1.687	1.645	1.484	1.944	1.924	1.908	1.895	1.838
LE (*D*_1_)	640.236	259.627	172.729	130.386	53.893	**1.439**	**1.152**	**0.977**	**0.859**	**0.565**
LE (*D*_2_)	**1.437**	**1.155**	**0.984**	**0.874**	**0.587**	**1.439**	**1.152**	**0.977**	**0.859**	**0.565**
LE (*D*_3_)	6.649	3.124	2.185	1.653	0.733	**1.439**	**1.152**	**0.977**	**0.859**	**0.565**
LE (*D*_4_)	220.533	129.204	102.887	87.006	45.331	**1.439**	**1.152**	**0.977**	**0.859**	**0.565**
LE (*D*_5_)	**1.437**	**1.155**	**0.984**	**0.872**	**0.587**	**1.439**	**1.152**	**0.977**	**0.859**	**0.565**

*ρ*=0.999										
MLE	22798.240	6332.474	3651.496	2566.870	926.151	196357.100	5573.940	3233.031	2278.584	826.081
RE (*k*_2_)	1.506	1.250	1.092	0.986	0.713	1.849	1.765	1.712	1.676	1.584
RE (*k*_7_)	1.532	1.244	1.050	0.913	0.527	1.425	1.130	0.945	0.819	0.471
RE (*k*_12_)	1.928	1.902	1.884	1.867	1.796	1.993	1.974	1.968	1.964	1.944
LE (*D*_1_)	6415.618	2567.391	1699.215	1278.986	521.731	**1.425**	**1.130**	**0.945**	**0.818**	**0.470**
LE (*D*_2_)	**1.425**	**1.134**	**0.955**	**0.834**	**0.501**	**1.425**	**1.130**	**0.945**	**0.818**	**0.470**
LE (*D*_3_)	53.531	20.366	12.527	8.245	1.884	**1.425**	**1.130**	**0.945**	**0.818**	**0.470**
LE (*D*_4_)	2158.873	1257.337	998.371	842.321	434.501	**1.425**	**1.130**	**0.945**	**0.818**	**0.470**
LE (*D*_5_)	**1.425**	**1.134**	**0.955**	**0.834**	**0.501**	**1.425**	**1.130**	**0.945**	**0.818**	**0.470**

*ρ*=0.9999										
MLE	227775.300	63256.020	36477.080	25641.810	9251.13	196357.100	55676.490	32295.150	22760.94	8251.422
RE (*k*_2_)	1.505	1.249	1.091	0.985	0.712	1.849	1.765	1.712	1.676	1.584
RE (*k*_7_)	1.532	1.244	1.049	0.912	0.525	1.425	1.128	0.942	0.814	0.461
RE (*k*_12_)	1.976	1.967	1.961	1.955	1.930	1.993	1.991	1.989	1.988	1.981
LE (*D*_1_)	63840.260	25673.520	16963.610	12777.610	5199.849	**1.425**	**1.127**	**0.941**	**0.813**	**0.460**
LE (*D*_2_)	**1.424**	**1.132**	**0.952**	**0.830**	**0.492**	**1.425**	**1.127**	**0.941**	**0.813**	**0.460**
LE (*D*_3_)	522.672	192.977	116.222	74.421	14.028	**1.425**	**1.127**	**0.941**	**0.813**	**0.460**
LE (*D*_4_)	21531.760	12537.490	9952.961	8395.063	4325.936	**1.425**	**1.127**	**0.941**	**0.813**	**0.460**
LE (*D*_5_)	**1.424**	**1.132**	**0.952**	**0.830**	**0.492**	**1.425**	**1.127**	**0.941**	**0.813**	**0.460**

*Note*. RE (*a*) indicates the ridge estimator based on the ridge parameter estimator a, LE (*b*) indicates the Liu estimator based on the Liu parameter estimator *b*, and bold values indicate the minimum SMSEs.

**Table 8:** Estimated SMSE values for different *n*, *ρ*, *k*, *d*,  and *β*_0_ when *δ*=0.04,  *α*=0.03,  and *p*=4.

*ρ*=0.99										
Estimator	*β*_0_=−1					*β*_0_=1				
	*n*=10	*n*=20	*n*=30	*n*=40	*n*=100	*n*=10	*n*=20	*n*=30	*n*=40	*n*=100
MLE	1290.178	351.061	201.273	141.149	50.739	1023.268	289.402	167.742	118.19	42.831
RE (*k*_2_)	1.278	1.001	0.854	0.764	0.559	1.770	1.675	1.623	1.592	1.520
RE (*k*_7_)	1.286	0.952	0.762	0.694	0.517	1.185	0.881	0.725	0.634	0.470
RE (*k*_12_)	1.667	1.544	1.468	1.405	1.184	1.893	1.854	1.827	1.802	1.706
LE (*D*_1_)	482.181	172.171	108.377	79.652	31.243	**1.183**	**0.879**	**0.723**	**0.632**	**0.469**
LE (*D*_2_)	**1.185**	**0.895**	**0.747**	**0.660**	**0.488**	**1.183**	**0.879**	**0.723**	**0.632**	**0.469**
LE (*D*_3_)	4.905	1.815	1.145	0.843	0.492	**1.183**	**0.879**	**0.723**	**0.632**	**0.469**
LE (*D*_4_)	227.801	109.915	78.862	62.425	28.354	**1.183**	**0.879**	**0.723**	**0.632**	**0.469**
LE (*D*_5_)	**1.185**	**0.895**	**0.747**	**0.660**	**0.488**	**1.183**	**0.879**	**0.723**	**0.632**	**0.469**

*ρ*=0.999										
MLE	12799.990	3474.189	1991.504	1396.258	501.591	10126.990	2863.031	1659.309	1169.029	423.504
RE (*k*_2_)	1.270	0.993	0.845	0.756	0.553	1.769	1.674	1.623	1.592	1.520
RE (*k*_7_)	1.279	0.941	0.749	0.628	0.352	1.162	0.841	0.667	0.558	0.300
RE (*k*_12_)	1.865	1.816	1.783	1.753	1.633	1.963	1.949	1.939	1.930	1.893
LE (*D*_1_)	4820.481	1696.644	1062.522	776.154	299.176	**1.161**	**0.840**	**0.666**	**0.557**	**0.299**
LE (*D*_2_)	**1.166**	**0.861**	**0.698**	**0.596**	**0.340**	**1.161**	**0.840**	**0.666**	**0.557**	**0.299**
LE (*D*_3_)	38.622	9.836	4.545	2.340	0.401	**1.161**	**0.840**	**0.666**	**0.557**	**0.299**
LE (*D*_4_)	2240.761	1069.345	763.603	602.223	268.874	**1.161**	**0.840**	**0.666**	**0.557**	**0.299**
LE (*D*_5_)	**1.166**	**0.861**	**0.698**	**0.596**	**0.340**	**1.161**	**0.840**	**0.666**	**0.557**	**0.299**

*ρ*=0.9999										
MLE	127910.100	34703.920	19894.650	13947.930	5010.152	101151.800	28598.560	16575.340	11677.740	4230.281
RE (*k*_2_)	1.270	0.992	0.844	0.755	0.552	1.769	1.674	1.623	1.592	1.520
RE (*k*_7_)	1.278	0.940	0.748	0.626	0.328	1.160	0.837	0.661	0.550	0.280
RE (*k*_12_)	1.953	1.936	1.924	1.913	1.865	1.988	1.983	1.980	1.977	1.964
LE (*D*_1_)	47952.080	16942.960	10592.630	7738.159	2977.766	**1.159**	**0.836**	**0.660**	**0.549**	**0.279**
LE (*D*_2_)	**1.164**	**0.857**	**0.693**	**0.589**	**0.323**	**1.159**	**0.836**	**0.660**	**0.549**	**0.279**
LE (*D*_3_)	375.952	90.268	39.045	17.907	0.908	**1.159**	**0.836**	**0.660**	**0.549**	**0.279**
LE (*D*_4_)	22376.190	10662.120	7610.758	5999.888	2673.596	**1.159**	**0.836**	**0.660**	**0.549**	**0.279**
LE (*D*_5_)	**1.164**	**0.857**	**0.693**	**0.589**	**0.323**	**1.159**	**0.836**	**0.660**	**0.549**	**0.279**

*Note*. RE (*a*) indicates the ridge estimator based on the ridge parameter estimator a, LE (*b*) indicates the Liu estimator based on the Liu parameter estimator *b*, and bold values indicate the minimum SMSEs.

**Table 9:** Estimated SMSE values for different *n*, *ρ*, *k*, *d*,  and *β*_0_ when *δ*=0.02,  *α*=0.25,  and *p*=4.

*ρ*=0.99										
Estimator	*β*_0_=−1					*β*_0_=1				
	*n*=10	*n*=20	*n*=30	*n*=40	*n*=100	*n*=10	*n*=20	*n*=30	*n*=40	*n*=100
MLE	1913.478	529.367	304.832	214.179	77.231	1615.252	457.738	265.474	187.103	67.842
RE (*k*_2_)	1.443	1.178	1.021	0.919	0.666	1.827	1.739	1.685	1.650	1.564
RE (*k*_7_)	1.465	1.160	0.964	0.832	0.583	1.365	1.067	0.894	0.782	0.524
RE (*k*_12_)	1.772	1.685	1.628	1.580	1.399	1.931	1.907	1.888	1.872	1.804
LE (*D*_1_)	590.911	229.175	150.091	112.173	45.497	**1.363**	**1.066**	**0.893**	**0.781**	**0.523**
LE (*D*_2_)	**1.362**	**1.071**	**0.904**	**0.798**	**0.545**	1.363	1.066	0.893	0.781	**0.523**
LE (*D*_3_)	6.187	2.681	1.803	1.335	0.613	**1.363**	**1.066**	**0.893**	**0.781**	**0.523**
LE (*D*_4_)	226.352	124.980	96.153	79.475	39.380	**1.363**	**1.066**	**0.893**	**0.781**	**0.523**
LE (*D*_5_)	**1.362**	**1.071**	**0.904**	**0.798**	**0.545**	**1.363**	**1.066**	**0.893**	**0.781**	**0.523**

*ρ*=0.999										
MLE	18970.200	5238.906	3016.275	2118.897	763.641	15980.020	4527.688	2625.743	1850.438	670.750
RE (*k*_2_)	1.437	1.170	1.013	0.910	0.659	1.826	1.738	1.685	1.650	1.564
RE (*k*_7_)	1.460	1.152	0.954	0.819	0.458	1.349	1.040	0.855	0.732	0.410
RE (*k*_12_)	1.912	1.881	1.859	1.838	1.754	1.976	1.968	1.961	1.955	1.931
LE (*D*_1_)	5905.543	2269.317	1474.808	1099.555	439.065	**1.348**	**1.039**	**0.855**	**0.731**	**0.409**
LE (*D*_2_)	**1.348**	**1.047**	**0.870**	**0.754**	**0.444**	**1.348**	**1.039**	**0.855**	**0.731**	**0.409**
LE (*D*_3_)	49.717	16.741	9.524	5.832	1.099	**1.348**	**1.039**	**0.855**	**0.731**	**0.409**
LE (*D*_4_)	2218.397	1216.301	932.605	768.820	376.437	**1.348**	**1.039**	**0.855**	**0.731**	**0.409**
LE (*D*_5_)	**1.348**	**1.047**	**0.870**	**0.754**	**0.444**	**1.348**	**1.039**	**0.855**	**0.731**	**0.409**

*ρ*=0.9999										
MLE	189540.800	52332.180	30131.600	21166.800	7627.807	159604.500	45226.060	26229.020	18484.280	6699.903
RE (*k*_2_)	1.436	1.169	1.012	0.910	0.658	1.826	1.738	1.684	1.650	1.564
RE (*k*_7_)	1.459	1.151	0.953	0.818	0.455	1.347	1.037	0.852	0.727	0.397
RE (*k*_12_)	1.970	1.960	1.952	1.945	1.914	1.992	1.989	1.987	1.985	1.977
LE (*D*_1_)	59285.760	22644.310	14721.380	10972.310	4374.427	**1.346**	**1.036**	**0.851**	**0.726**	**0.396**
LE (*D*_2_)	**1.346**	**1.044**	**0.867**	**0.749**	**0.432**	**1.346**	**1.036**	**0.851**	**0.726**	**0.396**
LE (*D*_3_)	485.473	157.635	87.075	51.146	6.811	**1.346**	**1.036**	**0.851**	**0.726**	**0.396**
LE (*D*_4_)	22132.520	12130.250	9296.824	7661.909	3746.686	**1.346**	**1.036**	**0.851**	**0.726**	**0.396**
LE (*D*_5_)	**1.346**	**1.044**	**0.867**	**0.749**	**0.432**	**1.346**	**1.036**	**0.851**	**0.726**	**0.396**

*Note*. RE (*a*) indicates the ridge estimator based on the ridge parameter estimator a, LE (*b*) indicates the Liu estimator based on the Liu parameter estimator *b*, and bold values indicate the minimum SMSEs.

**Table 10:** Estimated SMSE values for different *n*, *ρ*, *k*, *d*, and *β*_0_ when *δ*=0.04,  *α*=0.25,  and *p*=4.

*ρ*=0.99										
Estimator	*β*_0_=−1					*β*_0_=1				
	*n*=10	*n*=20	*n*=30	*n*=40	*n*=100	*n*=10	*n*=20	*n*=30	*n*=40	*n*=100
MLE	1203.887	326.347	186.925	131.032	47.070	942.456	266.410	154.391	108.775	39.413
RE (*k*_2_)	1.247	0.970	0.825	0.738	0.541	1.759	1.663	1.612	1.582	1.512
RE (*k*_7_)	1.251	0.915	0.728	0.644	0.490	1.152	0.849	0.698	0.611	0.467
RE (*k*_12_)	1.645	1.514	1.434	1.369	1.142	1.884	1.842	1.813	1.786	1.684
LE (*D*_1_)	465.044	162.790	102.135	74.654	29.225	**1.149**	**0.847**	**0.696**	**0.609**	**0.465**
LE (*D*_2_)	**1.153**	**0.865**	**0.722**	**0.640**	**0.482**	**1.149**	**0.847**	**0.696**	**0.609**	**0.465**
LE (*D*_3_)	4.658	1.685	1.056	0.783	0.484	**1.149**	**0.847**	**0.696**	**0.609**	**0.465**
LE (*D*_4_)	226.525	106.636	75.619	59.457	26.668	**1.149**	**0.847**	**0.696**	**0.609**	**0.465**
LE (*D*_5_)	**1.153**	**0.865**	**0.722**	**0.640**	**0.482**	**1.149**	**0.847**	**0.696**	**0.609**	**0.465**

*ρ*=0.999										
MLE	11945.500	3229.547	1849.498	1296.132	465.298	9327.907	2635.654	1527.277	1075.930	389.720
RE (*k*_2_)	1.239	0.961	0.816	0.729	0.535	1.758	1.663	1.612	1.582	1.512
RE (*k*_7_)	1.244	0.904	0.714	0.596	0.333	1.127	0.806	0.635	0.529	0.284
RE (*k*_12_)	1.854	1.801	1.766	1.734	1.606	1.960	1.944	1.934	1.923	1.883
LE (*D*_1_)	4607.413	1604.938	999.657	728.579	279.071	**1.126**	**0.805**	**0.635**	**0.529**	**0.283**
LE (*D*_2_)	**1.132**	**0.829**	**0.670**	**0.571**	**0.325**	**1.126**	**0.805**	**0.635**	**0.529**	**0.283**
LE (*D*_3_)	36.507	8.798	3.902	1.946	0.357	**1.126**	**0.805**	**0.635**	**0.529**	**0.283**
LE (*D*_4_)	2227.965	1037.162	731.880	573.204	252.443	**1.126**	**0.805**	**0.635**	**0.529**	**0.283**
LE (*D*_5_)	**1.132**	**0.829**	**0.670**	**0.571**	**0.325**	**1.126**	**0.805**	**0.635**	**0.529**	**0.283**

*ρ*=0.9999										
MLE	119374.600	32260.090	18476.060	12947.690	4647.611	93171.490	26327.380	15256.460	10747.770	3892.826
RE (*k*_2_)	1.238	0.961	0.816	0.729	0.535	1.757	1.663	1.612	1.582	1.512
RE (*k*_7_)	1.243	0.903	0.713	0.594	0.309	1.124	0.802	0.629	0.521	0.262
RE (*k*_12_)	1.949	1.930	1.918	1.905	1.854	1.987	1.981	1.978	1.974	1.961
LE (*D*_1_)	46079.010	16015.790	9967.305	7252.673	2774.694	**1.123**	**0.801**	**0.628**	**0.520**	**0.261**
LE (*D*_2_)	**1.130**	**0.825**	**0.665**	**0.564**	**0.307**	**1.123**	**0.801**	**0.628**	**0.520**	**0.261**
LE (*D*_3_)	354.078	80.259	32.898	14.224	0.620	**1.123**	**0.801**	**0.628**	**0.520**	**0.261**
LE (*D*_4_)	22260.380	10340.780	7294.258	5710.370	2509.670	**1.123**	**0.801**	**0.628**	**0.520**	**0.261**
LE (*D*_5_)	**1.130**	**0.825**	**0.665**	**0.564**	**0.307**	**1.123**	**0.801**	**0.628**	**0.520**	**0.261**

*Note*. RE (*a*) indicates the ridge estimator based on the ridge parameter estimator a, LE (*b*) indicates the Liu estimator based on the Liu parameter estimator *b*, and bold values indicate the minimum SMSEs.

**Table 11:** The estimated regression coefficients, standard errors (in parentheses), SMSE values, and AIC values of different Liu regression models for the simulated data set.

Parameter	LE_(*PMQL*)_ (SE)	LE_(*NB*)_ (SE)	LE_(Poisson)_ (SE)
$\hat{\beta_{0}}$	0.058 (0.123)	0.072 (0.037)	0.185 (0.028)
$\hat{\beta_{1}}$	0.517 (0.101)	0.453 (0.377)	0.291 (0.427)
$\hat{\beta_{2}}$	0.519 (0.101)	0.652 (0.382)	1.148 (0.426)
$\hat{\beta_{3}}$	0.518 (0.107)	0.550 (0.391)	0.075 (0.428)
$\hat{\beta_{4}}$	0.518 (0.108)	0.432 (0.395)	0.483 (0.425)
SMSE	**0.056**	0.600	0.729
AIC	**1168.221**	1292.462	6101.385

*Note*. Bold values indicate the minimum SMSE and AIC.

**Table 12:** The estimated regression coefficients, standard errors (in parentheses), SMSE values of MLE, and Liu estimator in PMQL regression model for the real data set.

Parameter	MLE_(*PMQL*)_ (SE)	LE_(PMQL)_ (SE)
$\hat{\beta_{0}}$	−1.046 (0.180)	−1.010 (0.165)
$\hat{\beta_{1}}$	0.408 (0.235)	0.396 (0.216)
$\hat{\beta_{2}}$	0.408 (0.076)	0.410 (0.075)
$\hat{\beta_{3}}$	0.407 (0.351)	0.441 (0.285)
$\hat{\beta_{4}}$	0.408 (0.090)	0.414 (0.088)
$\hat{\beta_{5}}$	0.408 (0.368)	0.375 (0.302)
$\hat{\beta_{6}}$	0.407 (0.101)	0.394 (0.099)
SMSE	0.371	**0.271**

*Note*. Bold value indicates the minimum SMSE.

## Data Availability

The data set is available for the public at https://www.football-data.co.uk/sweden.php. The data are also available from the author upon request.

## Appendix

### A. Lemma

Lemma 1 A.1. Let *M* be a positive definite (pd) matrix and *c* be a vector of nonzero constants. Then, *M* − *cc*′ > 0 iff *c*′*M*^−1^*c* < 1 [42].
